# A Photothermal‐Responsive PRP‐Loaded Hydrogel Engineered With Composite Nanobottle for Controllable Delivery of Growth Factors and Multifunctional Therapy of Diabetic Wounds

**DOI:** 10.1002/advs.202522427

**Published:** 2026-02-24

**Authors:** Wen‐Qiang Qu, Hui‐Yun Gu, Si‐Min Zeng, Chang‐Jiang Liu, Xiao Yan, Ting Pan, Chao Jian, Xian‐Zheng Zhang, Ai‐Xi Yu

**Affiliations:** ^1^ Department of Orthopedic Trauma and Microsurgery Zhongnan Hospital of Wuhan University Wuhan P. R. China; ^2^ Key Laboratory of Biomedical Polymers of Ministry of Education Department of Chemistry Wuhan University Wuhan P. R. China

**Keywords:** diabetic wound, multifunctionality, nanobottle, platelet‐rich plasma, photothermal response

## Abstract

In this paper, we develop a photothermal‐responsive hydrogel (CNB‐ePRP) based on engineered platelet‐rich plasma (PRP)/sodium alginate (SA) hydrogel integrating thrombin‐functionalized composite nanobottle (CNB) to treat diabetic wounds. Within CNB‐ePRP, the CNB consists of polydopamine nanobottle loaded with thrombin, personalized drugs and a phase‐change material. As a photothermal agent and dual‐stage controller, CNB not only enables in situ PRP activation at physiological temperature to slowly release various growth factors (GFs) via thermal‐triggered thrombin release, avoiding premature burst release of GFs, but also accelerates the release of GFs from the CNB‐ePRP hydrogel through mild photothermal heating under 808 nm laser irradiation. Meanwhile, SA enhances hydrogel stability and prolongs GF release kinetics. Consequently, CNB‐ePRP achieves on‐demand delivery of GFs, significantly promoting angiogenesis, cell proliferation and M2 macrophage polarization. Moreover, CNB‐ePRP can mitigate oxidative stress via the antioxidant activity of CNB. Furthermore, vancomycin is loaded into CNB to construct a multimodal antimicrobial hydrogel (VCNB‐ePRP) that combines antibiotic and photothermal therapy to efficiently eradicate MRSA and promote healing in MRSA‐infected diabetic mouse wounds. Collectively, this study offers a smart PRP‐derived platform, characterized by in situ PRP activation, on‐demand GFs release, personalized antimicrobial loading and multifunctional therapy, for managing diabetic wounds with great translational potential.

## Graphical abstract

A photothermal‐responsive engineered platelet‐rich plasma hydrogel (CNB‐ePRP) incorporated with thrombin‐functionalized composite nanobottle was developed, which enables in situ PRP activation, on‐demand growth factor release, multifunctionality, and personalized antimicrobial loading for treatment of diabetic wounds. The CNB‐ePRP hydrogel demonstrated remarkable curative effects and systematic therapeutic properties as validated in multiple diabetic wound mouse models.

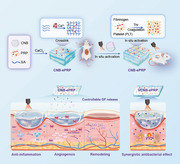

## 1 Introduction

Diabetes is a systemic metabolic disorder characterized by hyperglycemia and affects an estimated 783 million individuals worldwide by 2045 [1, 2]. Notably, nearly 25% of diabetic patients develop chronic nonhealing wounds, putting 33% of these patients at risk of amputation [3]. The prolonged treatment duration and high costs associated with diabetic wounds impose substantial financial burdens on both patients and healthcare systems [4, 5]. Multiple interrelated pathological factors present major challenges for diabetic wound repair [6]. Impaired neovascularization induced by hyperglycemic metabolism leads to severe perfusion deficiency and hypoxia, subsequently triggering excessive production of reactive oxygen species (ROS) [7, 8, 9]. Enhanced ROS generation causes oxidative damage, creating additional obstacles to angiogenesis and cellular proliferation. Meanwhile, persistent inflammation constitutes another critical barrier to healing [10]. Macrophages recruited to diabetic wounds preferentially polarize toward the proinflammatory (M1) phenotype, which produces proinflammatory mediators, ROS and proteases, exacerbating tissue damage [11, 12]. Moreover, sustained hyperglycemia and hypoxia increase susceptibility to recurrent bacterial infections, resulting in nutrient deprivation and systemic inflammation that further delays healing [13, 14]. Unfortunately, conventional therapeutic strategies often fail to comprehensively regulate this complex pathologic microenvironment, resulting in unsatisfactory prognosis [15]. Therefore, designing a versatile therapeutic strategy with pro‐angiogenesis, ROS scavenging, anti‐inflammatory and antibacterial properties is urgently needed for effective diabetic wound management.

During physiological hemostasis, platelets and fibrinogen are activated by thrombin to form a localized clot that orchestrates sequential tissue regeneration [16, 17, 18]. Inspired by this process, platelet‐rich plasma (PRP), an autologous plasma derivative enriched with supraphysiological concentrations of platelets, has emerged as a promising therapeutic component for wound healing [19]. Upon activation, platelets release a myriad of growth factors (GFs), including vascular endothelial growth factor (VEGF), platelet‐derived growth factor (PDGF), transforming growth factor‐β (TGF‐β), and epidermal growth factor (EGF), which synergistically regulate angiogenesis, inflammation, and cellular proliferation [20, 21, 22, 23]. Importantly, PRP preserves the physiological composition and ratios of GFs, offering advantages in safety and regenerative potential compared with commercial GFs [24, 25]. These features render PRP a unique bioactive component for engineering regenerative biomaterials [26, 27]. However, PRP application remains limited by rapid GF degradation, poor mechanical properties, and weak antibacterial activity [28, 29, 30]. Notably, in most PRP‐derived hydrogels, PRP is activated ex situ by direct thrombin addition during fabrication, inevitably leading to uncontrolled burst release of GFs, early depletion of platelet bioactivity and limited adaptability to the dynamic regenerative demands of diabetic wounds. Recent advances have explored PRP‐loaded hydrogels with stimulus‐responsive or degradation‐controlled GF release, including systems triggered by light, temperature or pH [20, 31, 32]. Besides, some hydrogels incorporate photothermal therapy (PTT) with other therapies to achieve synergistic antibacterial effects and multifunctional therapy [33, 34, 35, 36, 37, 38, 39, 40]. However, these strategies typically regulate GF diffusion indirectly and fail to achieve in situ platelet activation, limiting their ability to fully recapitulate the physiological coagulation‐mediated healing cascade. Consequently, bioengineered smart PRP‐derived hydrogels with in situ PRP activation, controllable GF release and highly synergistic multifunctionality remain unprecedented for the management of diabetic wounds.

**SCHEME 1:**
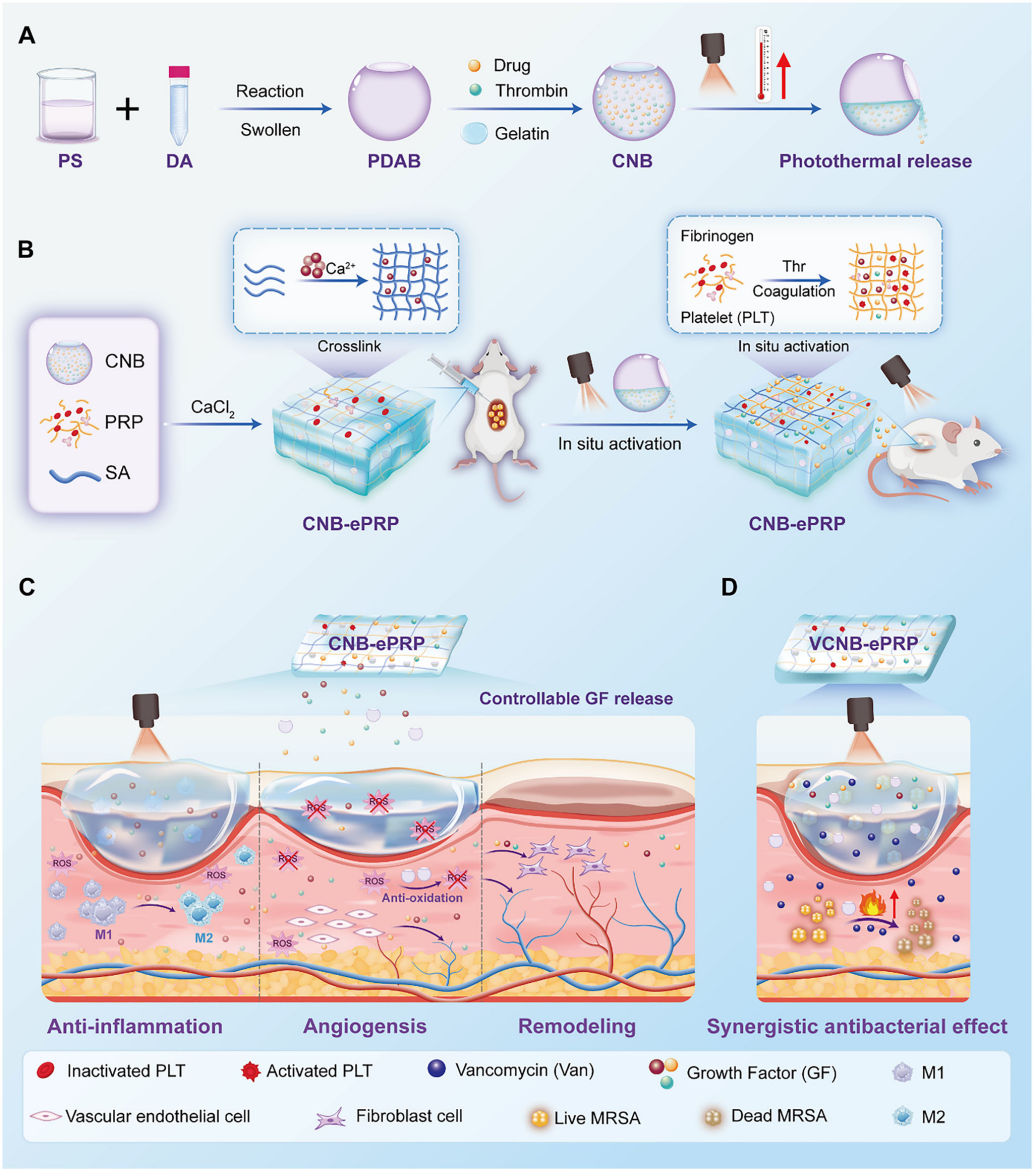
Schematic presentation of NIR photothermal‐responsive engineered PRP hydrogel (CNB‐ePRP) for accelerating diabetic wound and infected diabetic wound repair. (A) Preparation of thrombin‐functionalized composite nanobottle (CNB) via polydopamine nanobottle (PDAB) loading with thrombin, personalized drug and gelatin. (B) Preparation of injectable CNB‐ePRP and its mechanism of in situ activation of PRP and controllable release of GFs. (C) Biomedical application of CNB‐ePRP with multiple properties of pro‐angiogenesis, ROS scavenging, and anti‐inflammatory for the promotion of diabetic wound healing. (D) Expanded application of CNB‐ePRP by loading vancomycin (VCNB‐ePRP) with synergistic antibacterial capacity for promotion of MRSA‐infected diabetic wound repair.

To address these challenges, nanomaterial‐based controllers offer unique opportunities to regulate PRP bioactivity on demand. Among them, polydopamine (PDA) nanomaterials are attractive due to their outstanding near‐infrared (NIR) photothermal conversion efficiency, intrinsic antioxidant activity and facile structural/functional customization [41, 42, 43, 44]. In particular, PDA nanobottles (PDABs) featuring a hollow structure with defined surface openings enable ultrahigh payload loading, programmable release, surpassing conventional solid PDA nanoparticles [45, 46, 47]. Herein, inspired by the blood coagulation process, we elaborately designed a smart NIR photothermal‐responsive engineered PRP hydrogel (CNB‐ePRP) for diabetic wound treatment (Scheme 1A–D). This system is constructed by integrating thrombin‐functionalized composite nanobottle (CNB) into a PRP‐loaded sodium alginate (SA) hydrogel (ePRP) with in situ activated PRP, controllable release of GFs and multifunctions. Specifically, CNB is prepared by encapsulating thrombin, active drugs and a phase‐change material (PCM) within a PDAB, conferring photothermally controlled release capability. Upon injection into the wound site, CNB‐ePRP forms a dual‐network hydrogel via SA cross‐linking with Ca^2+^ and in situ PRP activation, closely mimicking the native coagulation microenvironment.

Notably, CNB functions as a multifunctional photothermal dual‐controller: (i) thermally releasing thrombin to activate PRP in situ, avoiding premature GF burst release; (ii) accelerating sustained GF secretion from the hydrogel under mild NIR irradiation. Meanwhile, the SA matrix further stabilizes the hydrogel and improves the sustained release kinetics, while the CNB provides antioxidant activity to scavenge ROS, thereby alleviating cellular oxidative stress. Consequently, CNB‐ePRP achieves continuous and controllable delivery of GFs, markedly enhancing angiogenesis and cell proliferation, while alleviating pathological inflammation by inducing anti‐inflammatory (M2) macrophage polarization. CNB‐ePRP demonstrates excellent therapeutic efficacy for diabetic wound healing in vivo. Furthermore, by loading vancomycin (Van) into CNBs, we extend this platform to a multimodal antibacterial hydrogel (VCNB‐ePRP) that combines vancomycin and PTT to eradicate methicillin‐resistant *Staphylococcus aureus* (MRSA) and accelerate the healing of MRSA‐infected diabetic wounds. Overall, the innovative CNB‐ePRP hydrogel integrates clinically‐approved PRP, in situ PRP activation, on‐demand GF release, multifunctional regulation and personalized antimicrobial loading, positioning it as a versatile strategy for diabetic wounds with high translational potential.

## 2 Results and Discussion

### 2.1 Synthesis and Characterization of CNB Nanoparticle

**FIGURE 1:**
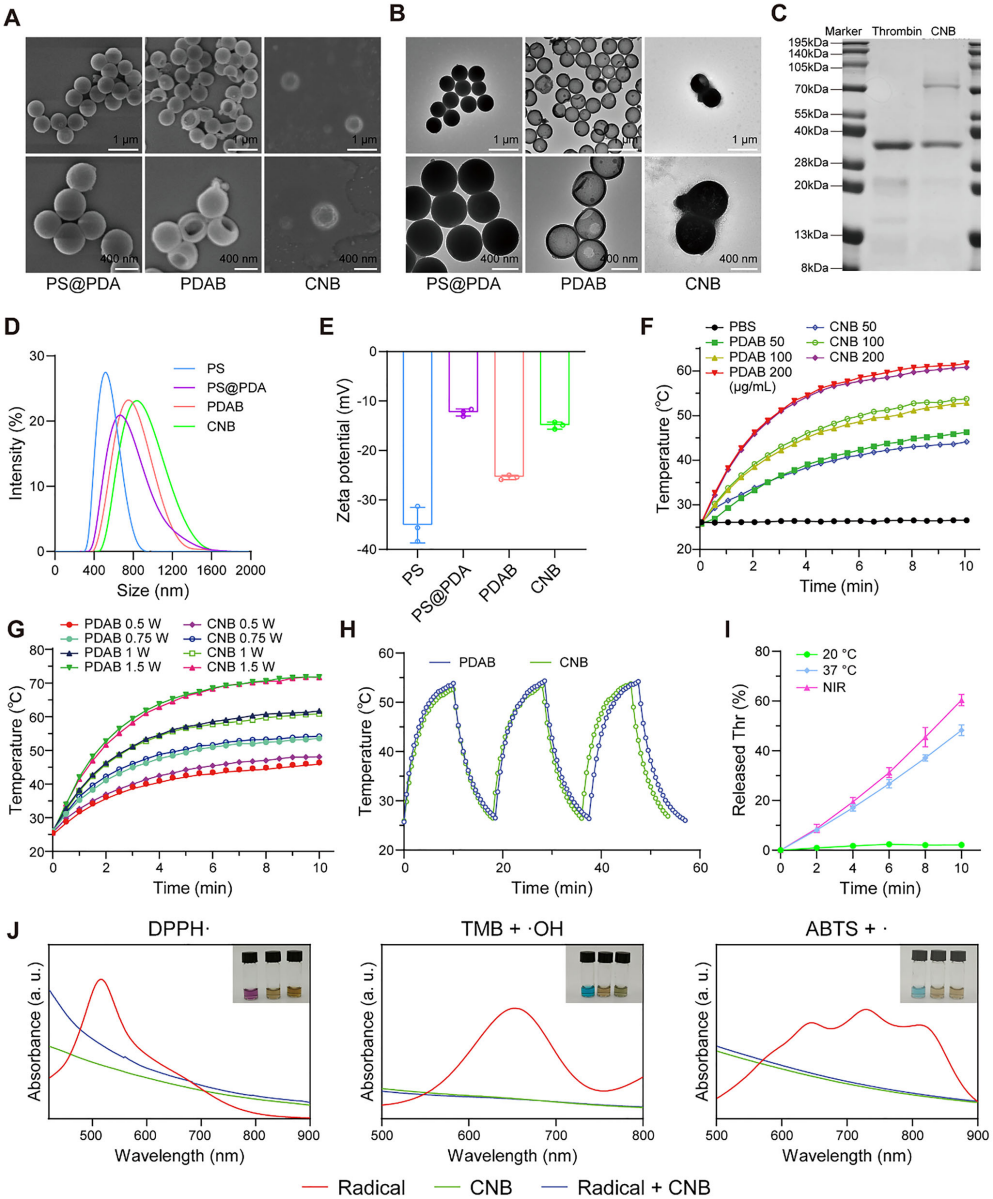
Characterization of PDAB and CNB nanoparticles. (A) SEM and (B) TEM images of PS@PDA, PDAB and CNB nanoparticles. (C) SDS‐PAGE analysis of CNB nanoparticles. (D, E) DLS and zeta potentials of PS, PS@PDA, PDAB and CNB nanoparticles. (F) Photothermal temperature curves of PDAB and CNB nanoparticles at different concentrations (0, 50, 100, 200 µg mL^−1^) under 808 nm laser irradiation (1.0 W cm^−2^) for 10 min (n = 3); (G) temperature curves of PDAB and CNB (200 µg mL^−1^) under 808 nm laser irradiation with different power densities (0.5, 0.75, 1.0, 1.5 W cm^−2^; n = 3). (H) Photothermal temperature cycling curves of PDAB and CNB (100 µg mL^−1^) under 808 nm laser irradiation (1.0 W cm^−2^; n = 3). (I) Thrombin release profiles from the CNB nanoparticles when incubated at 20°C, 37°C and 808 nm laser irradiation (1.0 W cm^−2^) within 10 min, respectively. (J) UV–Vis spectra of the radicals incubated with CNB nanoparticles. The insets are representative photographs of radicals only (Control), CNB and radicals + CNB groups arranged from left to right, respectively.

The fabrication process of thrombin‐based CNB started with the in situ polymerization of dopamine on polystyrene (PS) nanosphere surfaces, yielding PS@PDA core‐shell nanoparticles. Specifically, PDAB nanoparticles were perforated by ordinal expansion, extrusion, and dissolution of the PS core from the PS@PDA nanoparticles [48]. Analysis of scanning electron microscopy (SEM) and transmission electron microscopy (TEM) images (Figure 1A,B) confirmed that PDAB had a uniform diameter of 542.2 ± 24.6 nm and a hollow structure with surface open pores measuring 159.4 ± 69.1 nm. Subsequently, CNB was constructed by efficiently loading thrombin and a PCM composed of gelatin into the large internal space of PDAB through the pores. After removing the unloaded materials, SEM and TEM clearly showed that CNB was filled with solid contents. SDS‐PAGE analysis (Figure 1C) confirmed successful thrombin loading, as CNB exhibited a protein blot at approximately 37 kDa, consistent with free thrombin. The thrombin loading capacity of CNB was measured to be about 72.5 mg per gram of the PDAB. As expected, the hydrodynamic size of CNB increased to 870.9 ± 37.4 nm from 771.7 ± 54.5 nm of PDAB due to the loading of solid gelatin (Figure 1D). Zeta potential measurements indicated that CNB had a negative surface charge of ‐25.4 ± 0.5 mV, increasing from ‐15.0 ± 0.7 mV of PDAB (Figure 1E). These results confirmed the successful preparation of CNB. Furthermore, PDAB showed promising structural stability in PBS at 37°C during the one‐week observation, with minimal changes in hydrodynamic size and TEM structure (Figure S1A,B). By contrast, the hydrodynamic particle size of CNB increased with slight aggregation during storage in water at 4°C, while its TEM structure remained stable (Figure S1C,D).

PDA is a widely used photothermal agent with outstanding photothermal conversion ability upon 808 nm irradiation. Given that CNB contains PDAB, we evaluated its photothermal performance. The photothermal conversion effect of PDAB was positively correlated with CNB concentration and laser irradiation power. The heating rate of CNB was comparable to that of PDAB (Figure 1F,G; Figure S2). Under the irradiation of 808 nm laser (1 W cm^−2^), the 100 µg/mL of CNB solution increased to 48.2°C at 5 min and 53.8°C at 10 min, respectively. In addition, the photothermal effects of PDAB and CNB remained highly consistent over three on‐off cycles (Figure 1H), indicating high photothermal conversion efficiency. Considering PDAB's unique structural feature and photothermal property, the CNB holds great potential for NIR‐triggered controlled release and targeted drug delivery applications. Gelatin gel was employed as a smart PCM to entrap and modulate thrombin release due to its reversible gel‐sol transition properties around the upper critical solution temperature (UCST, ≈28‐35°C) [49, 50]. To this end, the thrombin release from CNB was evaluated under different conditions. As shown in Figure 1I, only 2.1% ± 0.5% of thrombin was released from CNB after 10 min at 20°C, indicating that gelatin was able to immobilize thrombin in PDAB. In contrast, when exposed to 37°C or 808 nm laser irradiation for the same time, CNB rapidly released 48.3% ± 2.2% and 60.4% ± 2.3% of thrombin, respectively. This burst release behavior was attributed to the gel–sol transition of the PCM, which rendered the matrix water‐soluble under both the conditions of 37°C and NIR laser irradiation. Taken together, these results demonstrate that CNB can be used for releasing thrombin in response to NIR laser irradiation.

PDA‐based nanomaterials are promising for scavenging ROS through a catechol/quinone redox system due to an abundance of unique phenolic structures. ROS primarily encompasses superoxide anion, ·OH, and hydrogen peroxide (H_2_O_2_) [51, 52]. The ROS scavenging capacity of CNB was evaluated by several free radical reaction assays [46], including the 1,1‐diphenyl‐2‐picryl‐hydrazyl radical (DPPH) assay, the tetramethylbenzidine (TMB) assay and the 2,2′‐azinobis‐3‐ethylbenzthiazoline‐6‐sulphonate (ABTS+·) assay (Figure 1J). Specifically, DPPH, TMB and ABTS+· exhibited specific colors and characteristic peaks at 517 nm, 652 nm and 734 nm in the presence of ROS, respectively. Upon addition of CNB, the DPPH solution faded from deep purple, with a corresponding reduction in its absorption peak at 517 nm. Similarly, the TMB solution and the ABTS+· solution also fade in color with diminished absorption peak intensities. The capacity to clear H_2_O_2_ was subsequently measured at different concentrations of CNB. The results (Figure S3) showed that the H_2_O_2_ concentration significantly decreased after incubation with 100 µg/mL CNB for 10 min. These results demonstrate the excellent capability of CNB to scavenge ROS and establish the foundation for cell protection under oxidative stress.

### 2.2 Construction and Characterization of CNB‐ePRP Hydrogel

**FIGURE 2:**
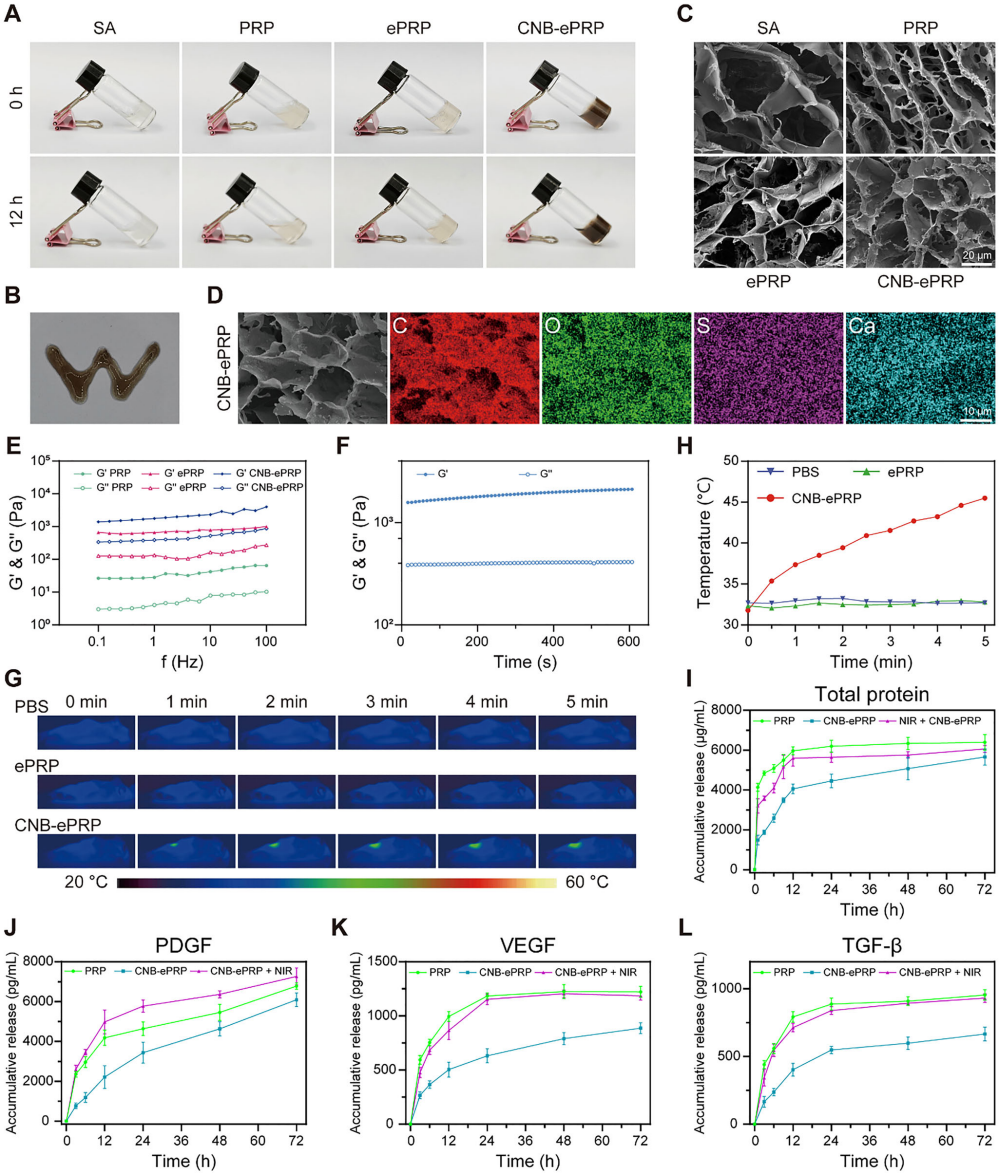
Characterization of CNB‐ePRP hydrogel. (A) Photographs of PRP, SA, ePRP, CNB‐ePRP at 0, 12 h. (B) Injection of the CNB‐ePRP hydrogel. (C) SEM images of PRP, SA, ePRP, CNB‐ePRP. (D) EDS elemental mapping of CNB‐ePRP. (E) Storage (G′) and loss (G″) modulus of PRP ePRP and CNB‐ePRP gels. (F) Storage (G′) and loss (G″) modulus of CNB‐ePRP gel under a time sweep condition. (G) Representative images of the photothermal temperature monitoring of CNB‐ePRP at mice wounds under an 808 nm laser irradiation (1.0 W cm^−2^) and (H) corresponding photothermal temperature curves (n = 3). (I) Total protein, (J) PDGF, (K) VEGF and (L) TGF‐β release profiles of CNB‐ePRP gel with or without NIR irradiation (1.0 W cm^−2^, 5 min, irradiation at 0, 1, 3, 6, 9, 12, 24 h; n = 4).

As illustrated in Scheme 1A, the photothermal‐responsive injectable CNB‐ePRP hydrogel was fabricated by integrating thrombin‐based CNB, FDA‐approved SA and clinically popular PRP. First, the engineered PRP (ePRP) solution was obtained by mixing 20 mg/mL of SA with PRP solution in a simple “one‐step” method. Subsequently, CNB‐ePRP hydrogel was obtained by gradually adding a mixture of CNB and calcium chloride into the ePRP solution. Within the CNB‐ePRP hydrogel, the SA solution rapidly forms “egg‐box” ionic crosslinked networks due to the reaction of carboxyl groups of the adjacent alginate backbone chains in the presence of Ca^2+^ [29]. On the other hand, thrombin and Ca^2+^ (especially thrombin) are key activators of PRP, and the slow release of thrombin from CNB activates the polymerization of fibrinogen monomers into a fibrin network. As shown in Figure 2A, the CNB‐ePRP dual‐network hydrogel rapidly formed a stable black hydrogel and maintained a homogeneously dispersed appearance even at 12 h post‐preparation. In contrast, the PRP single‐network gel immediately aggregated into clusters. Additionally, the prepared CNB‐ePRP hydrogel could be smoothly injected by a 0.45 mm diameter needle (Figure 2B; Figure S4). These results demonstrated that the addition of SA and calcium ions for cross‐linking could further stabilize the hydrogel network. From the perspective of clinical wound management, CNB‐ePRP hydrogel is more likely to adequately fill the wound tissue than PRP. We also added a mixture of CNB and calcium chloride to a simple PRP solution (CNB‐PRP), and CNB‐PRP was also able to form a gel as expected. Notably, CNB‐ePRP (Figure S5A,B) had a gelation time of about 13.3 min at 20°C, but rapidly formed a network hydrogel within 1.6 min at 37°C and even within 0.8 min under NIR irradiation (1 W cm^−2^). This result indicates that the internal gelation process of CNB‐ePRP involves the fibrous protein formed by PRP, and that CNB nanoparticles enable PRP to gelate in situ either at physiological temperature (about 37°C) or under NIR laser irradiation due to the thermally triggered release of thrombin from the CNB nanoparticles. SEM results confirmed that the CNB‐ePRP hydrogel had a representative porous three‐dimensional structure (Figure 2C). Moreover, energy dispersive spectroscopy (EDS) analysis (Figure 2D; Table S1) of the network elements further confirmed the presence of specific sulfur elements and uniformly distributed Ca elements, derived from self‐activated PRP, within the CNB‐ePRP gel. These findings provide additional evidence for an intrinsic fibronectin network embedded in the CNB‐ePRP hydrogel matrix. Rheological tests further confirmed the hydrogel structure and property. Under progressively increasing frequency, the storage modulus (G′) of all hydrogels surpassed the loss modulus (G″), confirming their dominant elastic behavior and stable gel‐like properties (Figure 2E). The poor mechanical properties of PRP, evidenced by a storage modulus (G′) of only 28.0 Pa at 1 Hz (Figure 2F), limit its clinical applicability. In contrast, ePRP and CNB‐ePRP gels exhibited significantly enhanced mechanical performance, with G′ values reaching 658.4 and 1771.1 Pa, respectively. Subsequently, we evaluated the in vitro simulated degradation capacity of different hydrogels. PRP hydrogel was completely degraded within 48 h in the culture medium, whereas CNB‐ePRP hydrogel exhibited a slow degradation profile with approximately 45.4% degraded at 72 h, demonstrating that CNB‐ePRP hydrogel has superior stability compared to PRP (Figure S6).

Photothermal performance of CNB‐ePRP is a critical feature to exert its regulatory function. To assess this, we evaluated the photothermal conversion capability of CNB‐ePRP in murine wound models (Figure 2G,H). Under 808 nm laser irradiation, the temperature of the wound site in the CNB‐ePRP group rapidly increased to 45.5°C within 5 min, comparable to that of CNB nanoparticles alone. In contrast, no significant temperature elevation was observed in the PBS or ePRP control groups. Notably, the temperature below 45°C represents a mild photothermal effect, which is generally safe in vivo applications with minimal risk of normal tissue damage [53, 54]. These results confirm that CNB‐ePRP maintains superior photothermal conversion efficiency in murine wound models while ensuring biosafety.

### 2.3 NIR‐Responsive Growth Factors Release From CNB‐ePRP Hydrogel

The capacity of PRP to deliver substantial quantities of GFs and bioactive proteins is crucial for regulating wound healing. However, primary limitations of PRP include rapid inactivation of GFs in the protease‐rich wound microenvironment and potential adverse effects resulting from excessively high local concentrations of bioactive components. Moreover, each phase of the wound healing process probably exhibits a specific demand for GF concentrations to effectively orchestrate cellular activities. To achieve optimal therapeutic outcomes, a sustained and controllable spatiotemporal release profile is essential to maintain appropriate GF concentrations in the diabetic wound microenvironment. To this end, the total protein release behavior of PRP gel and CNB‐ePRP gel was first monitored using the bicinchoninic acid (BCA) assay by measuring the absorbance at 562 nm. The results presented in Figure 2I confirmed that PRP gel exhibited a burst release profile, with the cumulative release rate reaching 75.8% at 3 h and further increasing to 93.4% at 12 h. Conversely, the CNB‐ePRP hydrogel demonstrated prolonged‐release profiles characterized by a relatively steady release rate, with the cumulative release amount reaching 29.4% at 3 h and increasing to 63.5% at 12 h. In addition, the cumulative total protein release from CNB‐ePRP gel was significantly higher at CNB concentrations of 100 µg/mL and 200 µg/mL than at 50 µg/mL (Figure S7), although the cumulative total protein release in the CNB concentration between 100 µg/mL and 200 µg/mL showed no significant difference. Notably, when added with NIR irradiation, CNB‐ePRP gel exhibited a faster release profile, where the release rate reached 56.1% at 3 h and increased to 87.6% at 12 h. These results indicate that CNB‐ePRP gel provides sustained release kinetics through its dense double network and holds the accelerated release property by NIR‐induced photothermal effects.

To further evaluate the release kinetics of bioactive proteins, we selected PDGF, VEGF, TGF‐β, and EGF as representative GFs and measured their release behaviors using an ELISA assay (Figure 2J–L; Figure S8). Interestingly, in the PRP gel, a burst release behavior of all these GFs was observed in the first few hours, similar to the total protein release profile, while the release rate was significantly slower in the CNB‐ePRP group. For example, the cumulative VEGF release rate from PRP gel reached 48.7% at 3 h and 81.3% at 12 h, whereas the VEGF release rate from CNB‐ePRP gel reached only 21.9% at 3 h and 41.3% at 12 h. As expected, significant acceleration of release behaviors of GFs (PDGF, VEGF, TGF‐β, and EGF) was also observed in the CNB‐ePRP + NIR group. The VEGF release rate from the CNB‐ePRP + NIR group also reached 39.0% at 3 h and increased to 70.9% at 12 h. The accumulative release of VEGF, TGF‐β and EGF in the PRP group and CNB‐ePRP + NIR group reached equilibrium at 24 h, and with no longer significant increase, while the release of PDGF continued to increase slightly. Such discrepancies in release kinetics are likely attributable to the distinct structural characteristics, concentration‐dependent effects, and binding affinities of the encapsulated GFs. Moreover, all GFs in CNB‐ePRP gel exhibited prolonged release behavior for at least 72 h. Collectively, these results demonstrated that CNB‐ePRP gel held excellent capabilities for continuous GFs release and NIR photothermal‐responsive control, which may offer potential for personalized on‐demand disease treatment.

### 2.4 Promotion of Proliferation and Angiogenesis Effect of CNB‐ePRP Hydrogel In Vitro

**FIGURE 3:**
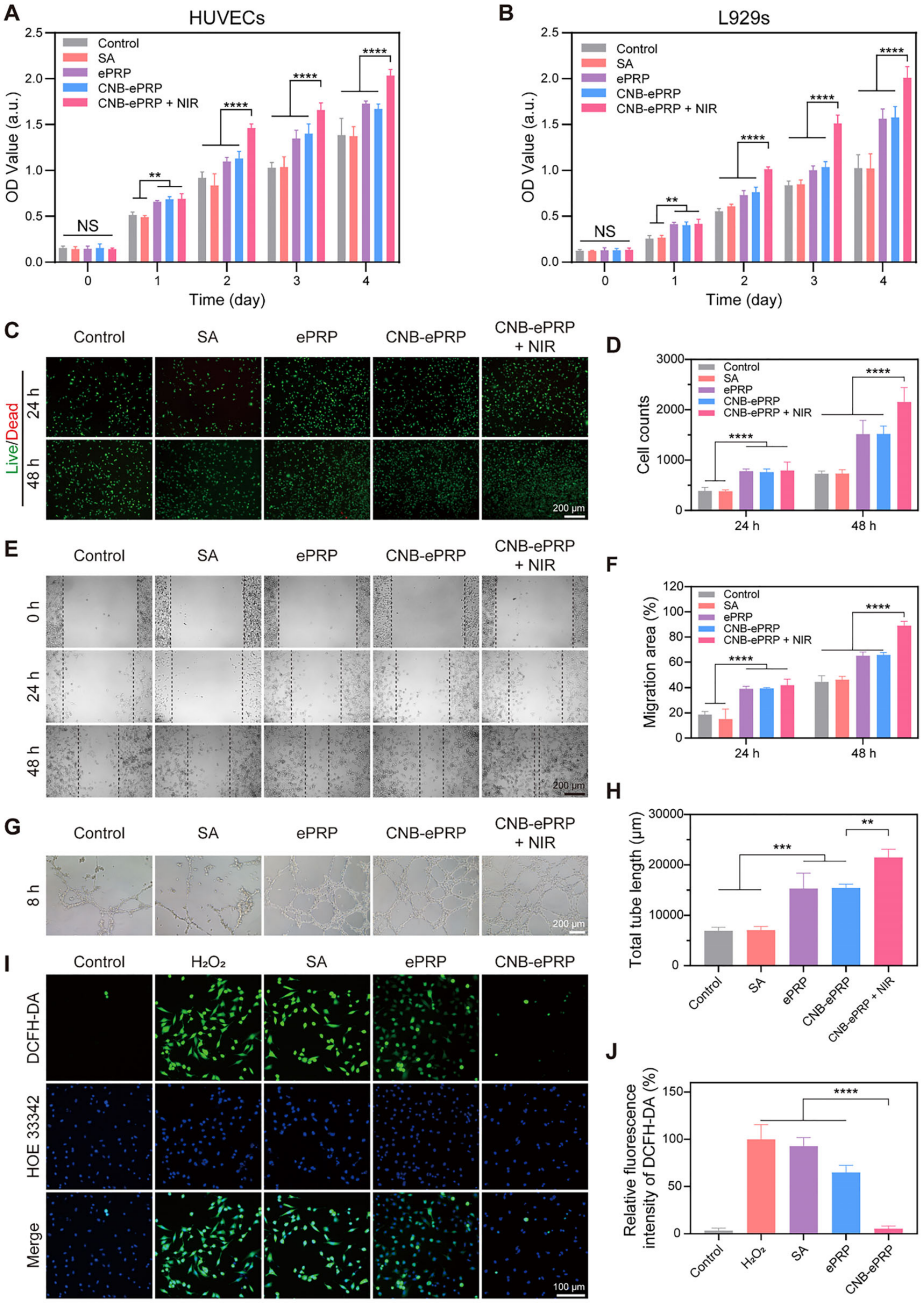
In vitro pro‐proliferation, pro‐angiogenesis and antioxidant activities of CNB‐ePRP gel. (A) Cell proliferation results of HUVECs cells treated with PBS, SA, ePRP, CNB‐ePRP, CNB‐ePRP + NIR, measured using the CCK‐8 assay; (B) Cell proliferation results of L929 cells (n = 6). (C) Representative fluorescence micrographs of live/dead staining for HUVECs cells after various treatments (at 24 and 48 h) and (D) quantification of the cell counts (n = 3). (E) Representative images of scratch assay for HUVECs cells and (F) quantification of the cell migration area (n = 3). (G) Representative micrographs of HUVECs tube formation assay after various treatments (at 8 h) and (H) quantification of the total tube length (n = 3). (I) Representative fluorescence images of intracellular ROS level for HUVECs in an oxidative stress environment (100 µM H_2_O_2_), stained by the ROS probe DCFH‐DA and the nuclear dye Hoechst 33342; (J) relative fluorescence intensity analysis of the DCFH‐DA probe (n = 3). Data are represented as means ± SD. Statistical significance is calculated by one‐way ANOVA with Tukey post hoc test. *p < 0.05, **p < 0.01, ***p < 0.001, ****p < 0.0001, NS means not significant.

The growth factors (such as VEGF, PDGF and EGF) in PRP are well‐established for their regenerative capabilities, particularly in promoting cell proliferation, migration, and angiogenesis [55, 56]. Given that CNB‐ePRP can modulate growth factor release kinetics under NIR irradiation, we investigated whether these bioactive properties are preserved in CNB‐ePRP gel. Considering that excessively high temperatures may impair growth factors’ bioactivity, we first selected VEGF as the representative to simulate and evaluate the effect of heating on the activity of growth factors encapsulated in the hydrogel. The results showed heating‐incubation at 45°C for 10 min had no significant effect on VEGF‐induced HUVEC proliferation activity (Figure S9), preliminarily demonstrating that the mild photothermal effect in this study did not significantly compromise growth factors’ bioactivity in CNB‐ePRP. Next, we examined the cytotoxicity of CNB nanoparticles in CNB‐ePRP gel on HUVECs and L929s cells using CCK‐8 assays. After 24 h of incubation, CNB at concentrations up to 200 µg/mL maintained cellular viability above 85%, demonstrating excellent biocompatibility (Figure S10A,B). To evaluate the bioactivity of growth factors released from different hydrogels, CCK‐8 proliferation assays were performed using HUVECs and L929s cells. As shown in Figure 3A,B, both HUVECs and L929s cells treated with ePRP or CNB‐ePRP displayed relatively higher proliferation rates on day 1 compared to control and SA gel groups, attributable to growth factor release following PRP activation. Notably, when CNB‐ePRP was subjected to NIR irradiation on day 1, the cells in the CNB‐ePRP + NIR group exhibited the most pronounced proliferative efficiency from day 2 to day 4, surpassing all other treatment groups. This enhanced proliferative effect primarily results from NIR photothermal‐triggered acceleration of growth factor release, consistent with our previous findings on NIR‐regulated growth factor release behaviors from CNB‐ePRP. Additionally, calcein‐AM staining (green fluorescence) confirmed significantly increased proliferation of HUVECs in the CNB‐ePRP + NIR group at 48 h (Figure 3C,D), providing further evidence for the photothermal‐enhanced bioactivity. Angiogenesis, which encompasses endothelial cell migration and de novo blood vessel formation, serves as a pivotal process in diabetic wound healing. In the scratch assay, we quantitatively compared endothelial cell migration rates after different treatments. As demonstrated in Figure 3E,F, CNB‐ePRP with NIR irradiation significantly enhanced the migration of HUVECs compared to other treatments. Furthermore, the results of tube formation assays were consistent with the scratch healing experiments, as CNB‐ePRP with NIR irradiation significantly enhanced the ability of HUVECs to form blood vessel structures (Figure 3G,H; Figure S11A,B). These in vitro experiments collectively demonstrated that: CNB‐ePRP effectively promotes cellular proliferation and vascular regeneration through its sustained release of GFs; NIR‐triggered acceleration of growth factor release further enhanced these pro‐regenerative effects.

### 2.5 Antioxidant Activity of CNB‐ePRP Hydrogel In Vitro

A hallmark of diabetic wounds is the presence of elevated ROS levels and persistent oxidative stress. Previous studies have demonstrated that incorporating antioxidant components into hydrogels can mitigate oxidative cellular damage and accelerate chronic wound healing [57, 58]. Given the well‐documented antioxidant properties of PDA, CNB‐ePRP hydrogel holds promise for promoting diabetic wound healing. To simulate the oxidative stress microenvironment of diabetic wounds, HUVECs were treated with 100 mM H_2_O_2_ in combination with different materials. Following a specific incubation period, intracellular ROS levels were assessed. H_2_O_2_ exposure induced a marked increase in green fluorescence intensity for HUVECs cells, reflecting substantial intracellular ROS accumulation (Figure 3I,J). Importantly, treatment with CNB‐ePRP hydrogel significantly attenuated this oxidative response, reducing intracellular ROS levels compared to other H_2_O_2_‐treated groups. In addition, cell activity was subsequently evaluated using the CCK‐8 assay following culture for 24 h. CNB‐ePRP hydrogel treatment maintained significantly higher cell viability compared to both the other H_2_O_2_‐treated groups and the normal control group (Figure S12). These results demonstrated that CNB‐ePRP hydrogel effectively scavenged intracellular ROS and protected against oxidative stress‐induced damage while promoting cellular proliferation.

### 2.6 Anti‐Inflammatory Activity of CNB‐ePRP Hydrogel In Vitro

**FIGURE 4:**
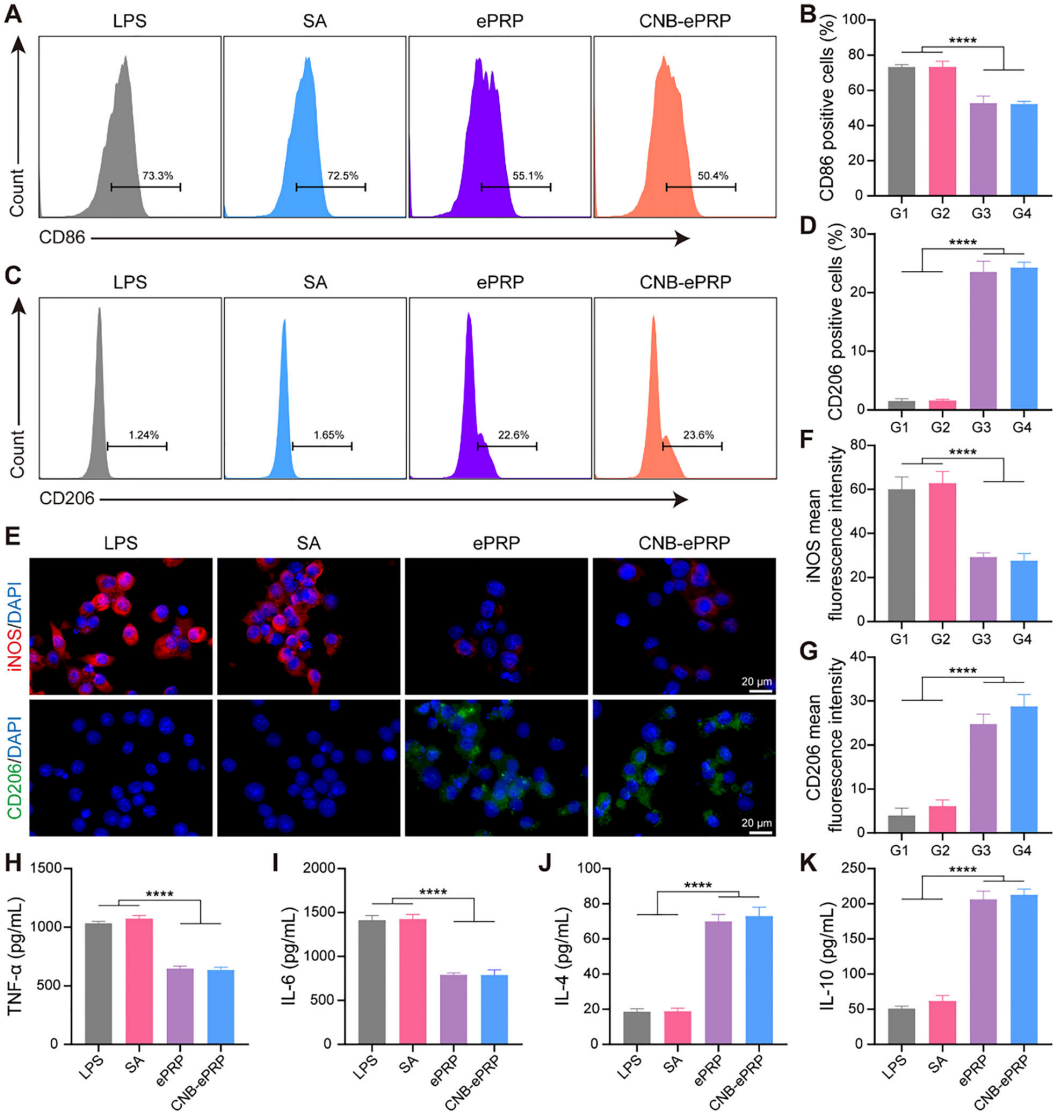
Anti‐inflammatory effects of CNB‐ePRP on macrophages. (A, B) The expression of M1 (CD86^+^) on RAW264.7 cells in various groups detected by flow cytometry and quantitative analysis for the percentage of M1 (CD86^+^) macrophages. (C, D) The expression of M2 (CD206^+^) on RAW264.7 cells by flow cytometry and quantitative analysis (n = 4). (E) Representative immunofluorescence micrographs for CD86 (M1 macrophage marker, red) and CD206 (M2 macrophage marker, green); (F, G) corresponding quantification of CD86 intensity and CD206 in RAW264.7 cells after 24 h of different treatments (n = 3). (H, I) TNF‐α, IL‐6 and (J, K) IL‐4, IL‐10 concentrations secreted from RAW264.7 cells after treatment, measured by ELISA (n = 4). Data are represented as means ± SD. Statistical significance is calculated by one‐way ANOVA with Tukey post hoc test. *p < 0.05, **p < 0.01, ***p < 0.001, ****p < 0.0001, NS means not significant.

Macrophages represent an important type of immune cell that functions through phagocytosis, cytotoxicity, and cytokine secretion. Among them, M2 macrophages are involved primarily in immune response modulation, tissue repair and regeneration. PRP is widely recognized for its ability to balance pro‐reparative and immune‐regulatory functions due to the release of high levels of growth factors and chemokines from the activated platelets [18, 55, 59]. For example, TGF‐β drives the polarization of M1 macrophages toward the M2, upregulating the secretion of anti‐inflammatory cytokines (Arg‐1, IL‐10) to resolve excessive inflammation. To determine whether CNB‐ePRP exhibits similar immunomodulatory effects, the polarization of RAW264.7 cells was investigated after treatment with LPS and different materials. Flow cytometer (FCM) analysis revealed a significant increase in CD86 expression in RAW264.7 cells after treating with LPS, while CD86 expression was markedly reduced in the ePRP group and CNB‐ePRP groups (Figure 4A,B). Conversely, CD206 expression was upregulated in the ePRP and CNB‐ePRP groups (Figure 4C,D). Additionally, changes in the M1/M2 ratio also indicated a shift from a proinflammatory to an anti‐inflammatory immune microenvironment (Figure S13). After treatment, immunofluorescence staining (IF) and corresponding quantitative analysis of iNOS and CD206 further supported the FCM results (Figure 4E–G). Moreover, ELISA results highlighted that the ePRP and CNB‐ePRP groups exhibited markedly lower levels of pro‐inflammatory cytokines, including tumor necrosis factor‐𝛼 (TNF‐𝛼), interleukin‐1𝛽 (IL‐1𝛽), and interleukin‐6 (IL‐6), along with a pronounced elevation in the expression of anti‐inflammatory cytokines (IL‐4 and IL‐10) compared to the LPS and SA groups (Figure 4H–K; Figure S14). Similarly, qPCR results (Figure S15A–D) revealed that the ePRP and CNB‐ePRP groups exhibited a lower expression level of pro‐inflammatory genes (TNF‐α, iNOS) and higher expression of anti‐inflammatory genes (Arg‐1, IL‐10). These results suggest that CNB‐ePRP efficiently shifts macrophage polarization from pro‐inflammatory to anti‐inflammatory phenotypes, reducing the expression levels of inflammatory genes and the secretion of inflammatory factors.

### 2.7 Accelerating Diabetic Wound Healing by CNB‐ePRP Hydrogel In Vivo

**FIGURE 5:**
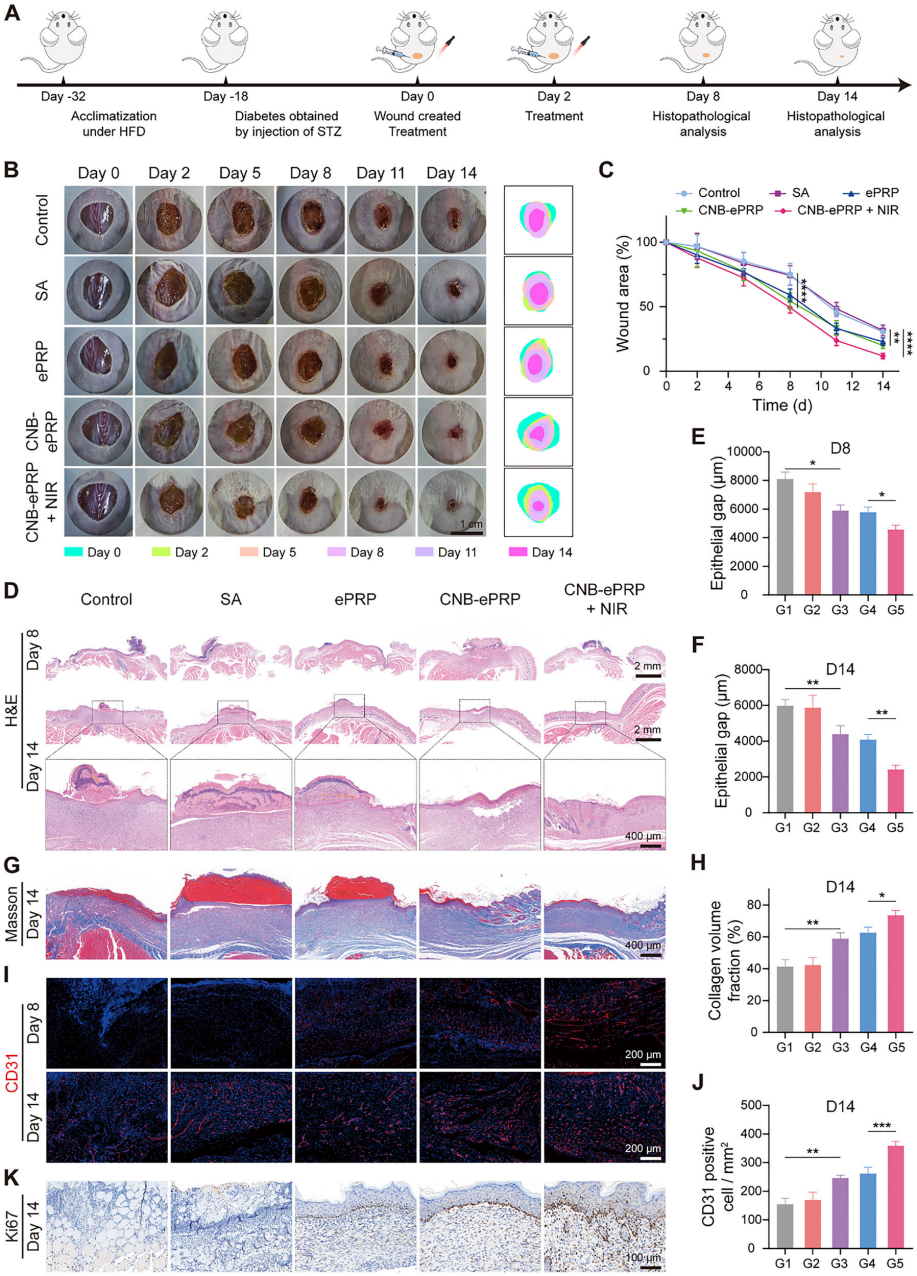
In vivo promotion of diabetic wound healing by CNB‐ePRP. (A) Schematic illustration for the establishment of the diabetic mice wound model and the experimental timeline. (B) Representative wound traces and simulations of mice in different groups and (C) corresponding analysis of wound contraction rate (n = 5). (D) Representative images of H&E staining of wound tissues and (E, F) damaged epithelial gap analysis on day 8 and day 14 post‐treatment, respectively (n = 3). (G) Representative images of Masson's staining of wound tissues and (H) quantitative analysis of collagen volume fractions (n = 3). (I) Representative IF images of CD31‐labeled cells for wound tissues and (J) quantitative analysis (n = 3). (K) Representative IHC images of Ki67 labels for wound tissues. Data are represented as means ± SD. Statistical significance is calculated by one‐way ANOVA with Tukey post hoc test. *p < 0.05, **p < 0.01, ***p < 0.001, ****p < 0.0001, NS means not significant.

The therapeutic approach is illustrated in Figure 5A. The successful modeling of diabetic mice was evidenced by the continuous increase in blood glucose value (Figure S16). Diabetic wounds with a 10 mm diameter were created, and different treatments were administered to the wound sites day 0 and day 2. In the CNB‐ePRP + NIR group, each topical application of CNB‐ePRP hydrogel was followed by 808 nm laser irradiation (1 W cm^−2^, 5 min). First, wound healing progression was monitored by a camera. Figure 5B,C presented macroscopic images of the wound closure and corresponding quantitative healing rates at sequential time points. On day 5, the CNB‐ePRP + NIR group exhibited markedly reduced wound areas compared to the control group. By day 8, the CNB‐ePRP + NIR group showed accelerated wound closure, whereas the wound sizes in the control, SA, ePRP and CNB‐ePRP gel groups remained at 75.1%, 74.3%, 59.0%, and 54.4%, respectively. On day 14, the CNB‐ePRP + NIR group achieved nearly complete wound closure, whereas the control and SA groups retained substantial unhealed areas at 30.6% and 31.6%, respectively. Notably, both ePRP and CNB‐ePRP groups displayed enhanced healing compared to controls, though less pronounced than the CNB‐ePRP + NIR group. These findings demonstrated that combined CNB‐ePRP hydrogel and 808 nm laser treatment significantly accelerated diabetic wound healing.

Histological evaluation of wounds was performed using hematoxylin‐eosin (H&E) staining (Figure 5D–F; Figure S17A). Analysis revealed a significantly reduced epidermal gap in the CNB‐ePRP + NIR group relative to other groups on both day 8 and day 14 post‐treatment. Although all groups demonstrated evidence of epidermal regeneration, the CNB‐ePRP + NIR group exhibited more physiologically mature tissue architecture with superior structural organization. Furthermore, the wound treated with CNB‐ePRP + NIR exhibited greater epidermal thickness compared to other groups on day 14. Masson's trichrome staining confirmed enhanced collagen deposition in the CNB‐ePRP + NIR group, with a collagen volume fraction (CVF) of 73.7%, which was significantly higher than that of the CNB‐ePRP group and other groups (Figure 5G,H). These findings collectively indicated that CNB‐ePRP gel with NIR irradiation could accelerate both epidermal regeneration and collagen matrix formation during wound healing.

To elucidate the mechanisms underlying re‐epithelialization in diabetic wounds, neovascularization of wound tissues was evaluated through CD31 IF staining. IF analysis (Figure 5I,J; Figure S17B) revealed increased CD31‐positive vessels in the ePRP, CNB‐ePRP, and CNB‐ePRP + NIR groups compared to the control and SA gel groups on days 8 and 14. Notably, the CNB‐ePRP + NIR group exhibited the most pronounced CD31 expression. Additionally, immunohistochemical (IHC) analysis of Ki67, a nuclear marker of cellular proliferation, showed an enhanced proliferative activity in the CNB‐ePRP + NIR treatment group compared to other groups (Figure 5K). Therefore, the above results demonstrated that NIR‐regulated CNB‐ePRP hydrogel could promote the re‐epithelialization process through superior angiogenesis and stimulated cellular proliferation in vivo. This effect is attributed to sustained release of angiogenic GFs and NIR‐enhanced growth factor release from CNB‐ePRP gel, thereby accelerating the repair of diabetic wounds in mice.

**FIGURE 6:**
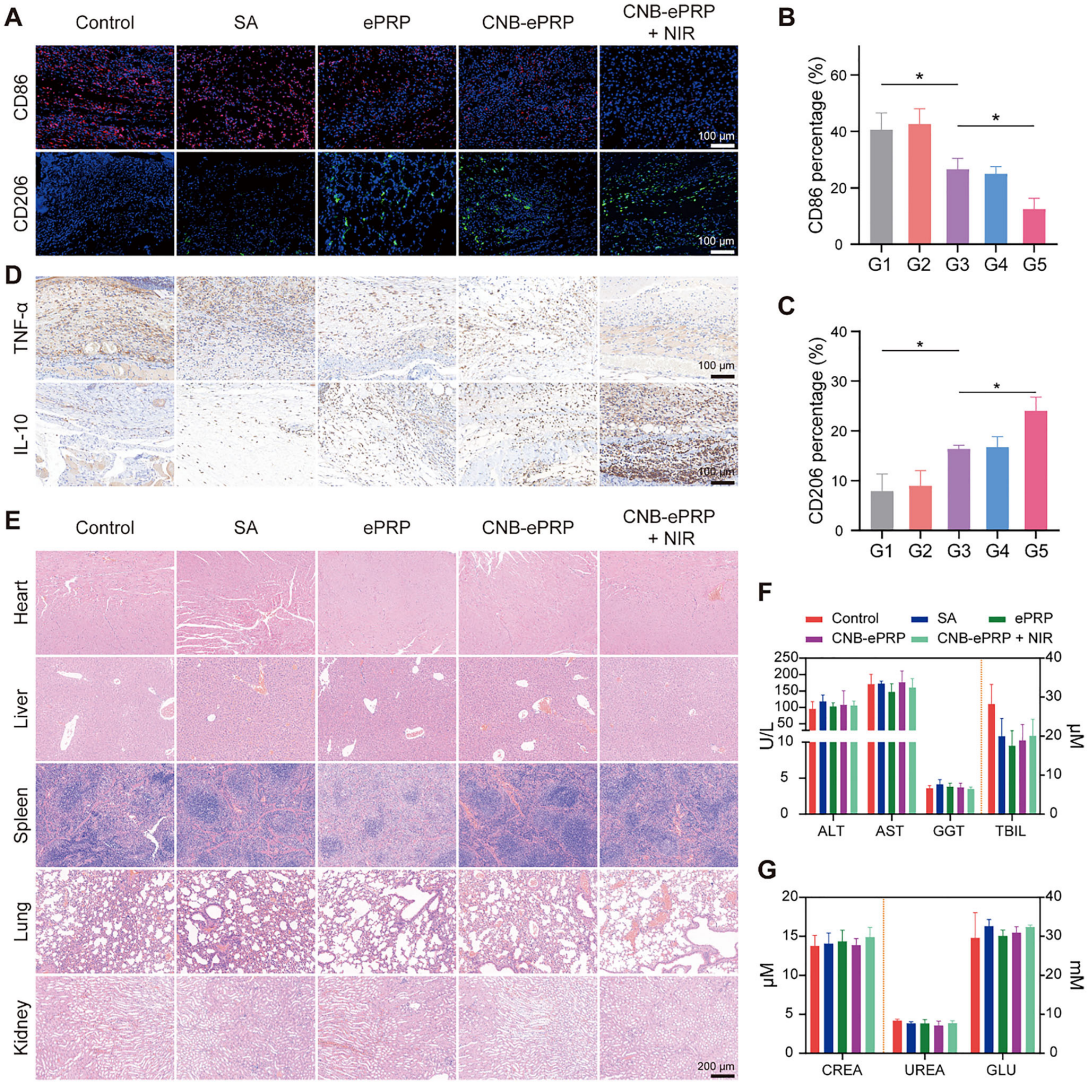
In vivo evaluation of immune regulation in diabetic wounds and biosafety. (A) Representative IF images of CD86‐labeled (red) and CD86‐labeled (green) cells for wound tissues and (B, C) corresponding quantitation analysis. (D) Representative IHC images of TNF‐α and IL‐10 for wound tissues. (E) Representative H&E staining images of major organs after treatment. (F, G) Biochemical parameters analysis. (n = 3). Data are represented as means ± SD. Statistical significance is calculated by one‐way ANOVA with Tukey post hoc test. *p < 0.05, **p < 0.01, ***p < 0.001, ****p < 0.0001, NS means not significant.

During diabetic wound repair, persistent macrophage‐associated hyperinflammation severely impairs subsequent vessel formation and skin regeneration. Therefore, an ideal therapeutic strategy should focus on reducing the levels of inflammation to facilitate repair. IF and IHC stainings were employed to assess the anti‐inflammatory capabilities of CNB‐ePRP gel for diabetic wound treatment. IF analysis of wound tissues (Figure 6A–C) revealed substantial infiltration of M2 macrophage (CD206 labeled) in the subcutaneous layer following CNB‐ePRP + NIR treatment, with significantly greater abundance compared to other groups. Conversely, a marked reduction in M1 macrophage (CD86‐labeled) populations was observed. In addition, IHC staining results (Figure 6D) showed the pro‐inflammatory cytokine (TNF‐α) expression decreased, with an increase of anti‐inflammatory cytokine (IL‐10) expression on day 8 following CNB‐ePRP + NIR treatment. These findings demonstrate that CNB‐ePRP + NIR treatment can alleviate the local inflammatory microenvironment of diabetic wounds, establishing a robust foundation for wound regeneration.

Finally, we evaluated the in vivo toxicity of different treatments through H&E staining of vital visceral tissues (heart, liver, spleen, lungs, and kidneys) and blood biochemical examinations. H&E staining showed that these organs maintained excellent integrity without significant damage or changes (Figure 6E). Furthermore, analysis of serum levels of alanine aminotransferase (ALT), gamma‐glutamyl transferase (GGT), aspartate aminotransferase (AST), total bilirubin (TBIL), creatinine (CREA) and blood urea nitrogen (UREA) revealed no significant differences among all treatment groups (Figure 6F,G). These results demonstrate that CNB‐ePRP hydrogel has excellent biocompatibility in vivo.

Overall, the CNB‐ePRP hydrogel showed remarkable potential in facilitating the repair of diabetic wounds by inhibiting inflammation and promoting neovascularization.

### 2.8 Synergistic Antibacterial Activity of VCNB‐ePRP In Vitro

**FIGURE 7:**
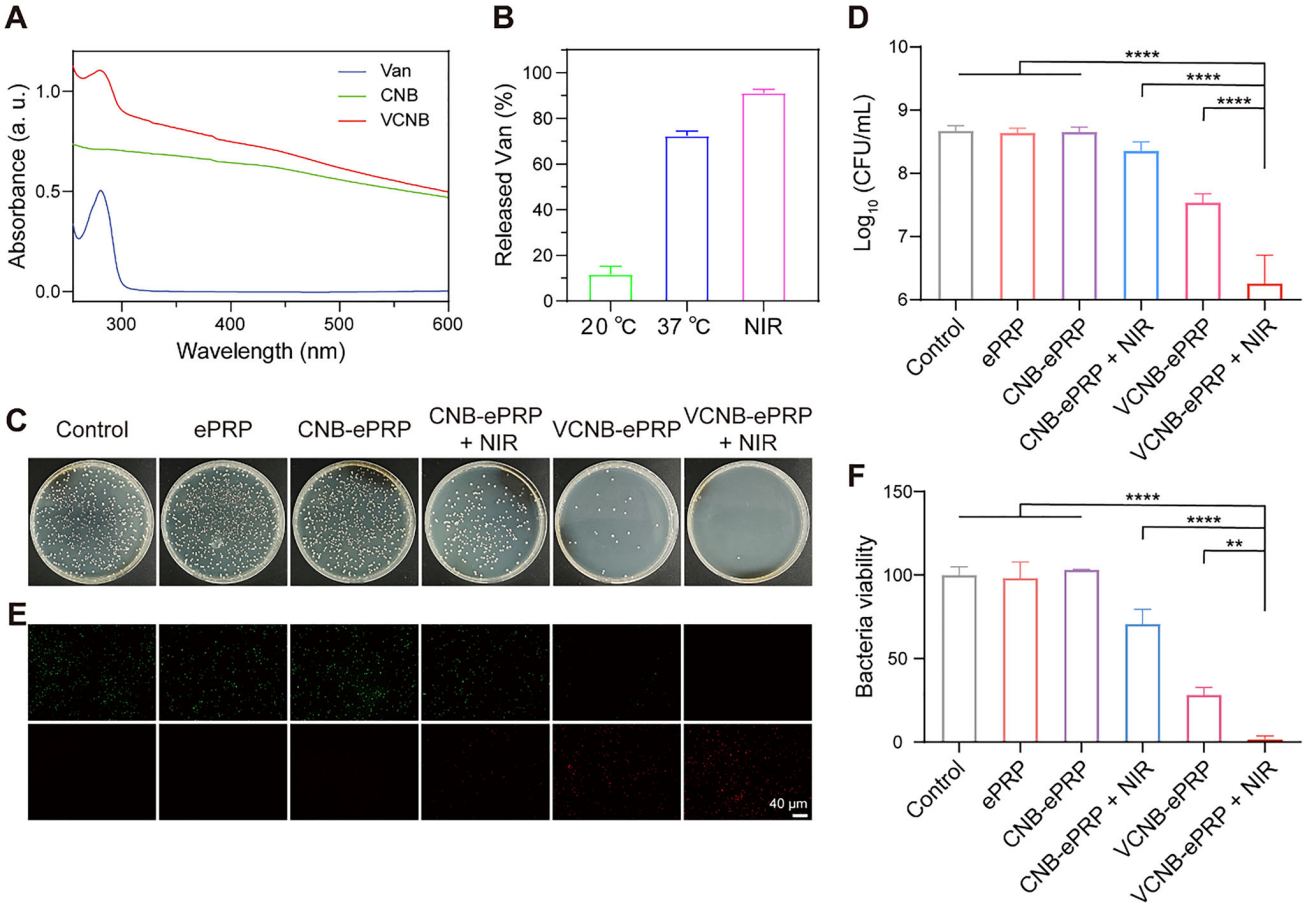
In vitro antibacterial activity of VCNB‐ePRP. (A) UV–vis spectroscopy analysis of VCNB nanoparticles. (B) Quantification analysis for NIR photothermal release of vancomycin from VCNB nanoparticles. (C) Representative images of survival MRSA clones on LB plates after treating with different materials (PBS donated as control, Van, ePRP, VCNB‐ePRP, VCNB‐ePRP) and (D) corresponding quantitation analysis (n = 3). (E) Images of the live/dead bacterial viability assay (scar bar: 100 µm) and (F) quantitative analysis of bacterial viability (n = 3). Data are represented as means ± SD. Statistical significance is calculated by one‐way ANOVA with Tukey post hoc test. *p < 0.05, **p < 0.01, ***p < 0.001, ****p < 0.0001, NS means not significant.

Diabetic wounds exhibit high susceptibility to bacterial infections and the global challenge of antibiotic resistance further complicates their antimicrobial management [60]. In view of the unique properties of CNB, including its high loading capacity and photothermal‐responsive release characteristics, we developed an expanded antimicrobial application of CNB‐ePRP. Specifically, vancomycin (a first‐line therapeutic drug against MRSA) was incorporated into CNB to construct VCNB and the corresponding VCNB‐ePRP hydrogel. UV–vis spectroscopy confirmed successful vancomycin loading in VCNB (Figure 7A). Notably, 808 nm laser irradiation significantly enhanced vancomycin release from VCNB and VCNB‐ePRP (Figure 7B; Figure S18), demonstrating the NIR photothermal‐responsive vancomycin release capability of this system.

To evaluate the antibacterial activity of VCNB‐ePRP, MRSA suspensions were incubated with VCNB‐ePRP with or without twice of 808 nm laser irradiation (1 W cm^−2^, 5 min; 10 min interval) and quantified bacterial viability through plate count analysis (Figure 7C,D). No significant bacterial death was observed in the Control, ePRP and CNB‐ePRP group, confirming that the CNB‐ePRP hydrogel lacks inherent bactericidal properties. When exposed to NIR irradiation, CNB‐ePRP hydrogel exhibited bactericidal efficiencies of 51.1% against MRSA, indicating the relatively modest effects under only mild photothermal conditions. This phenomenon is different from the direct and intense bactericidal activity of high‐temperature thermal ablation [34, 39]. Vancomycin can inhibit the formation of the peptidoglycan layer within the bacterial cell wall of Gram‐positive bacteria by binding to the precursor molecules and preventing normal intermolecular cross‐linking and polymerization reaction. Thus, VCNB‐ePRP even exhibited a bactericidal activity of 92.5% against MRSA with the sustained release of a low dose of vancomycin. Notably, upon exposure to NIR irradiation, VCNB‐ePRP underwent burst release of vancomycin, which acted synergistically with the photothermal effect, resulting in the VCNB‐ePRP +NIR group displaying superior bactericidal efficiencies of 99.4% against MRSA. As an effective antibiotic‐adjuvant therapy, NIR‐induced mild photothermal effects can disrupt bacterial peptidoglycan barrier integrity and enhance vancomycin permeability [36, 61, 62]. Our results are consistent with the antibacterial efficacy trends of materials combining vancomycin and mild photothermal therapy in previous studies [62, 63]. Correspondingly, SYTO‐9/PI staining assay confirmed minimal MRSA viability following VCNB‐ePRP + NIR treatment (Figure 7E,F). These findings established that VCNB‐ePRP achieved potent MRSA eradication in vitro through synergizing the mild otothermal effect and vancomycin activity.

### 2.9 Accelerating MRSA‐Infected Diabetic Wound Healing by VCNB‐ePRP In Vivo

**FIGURE 8:**
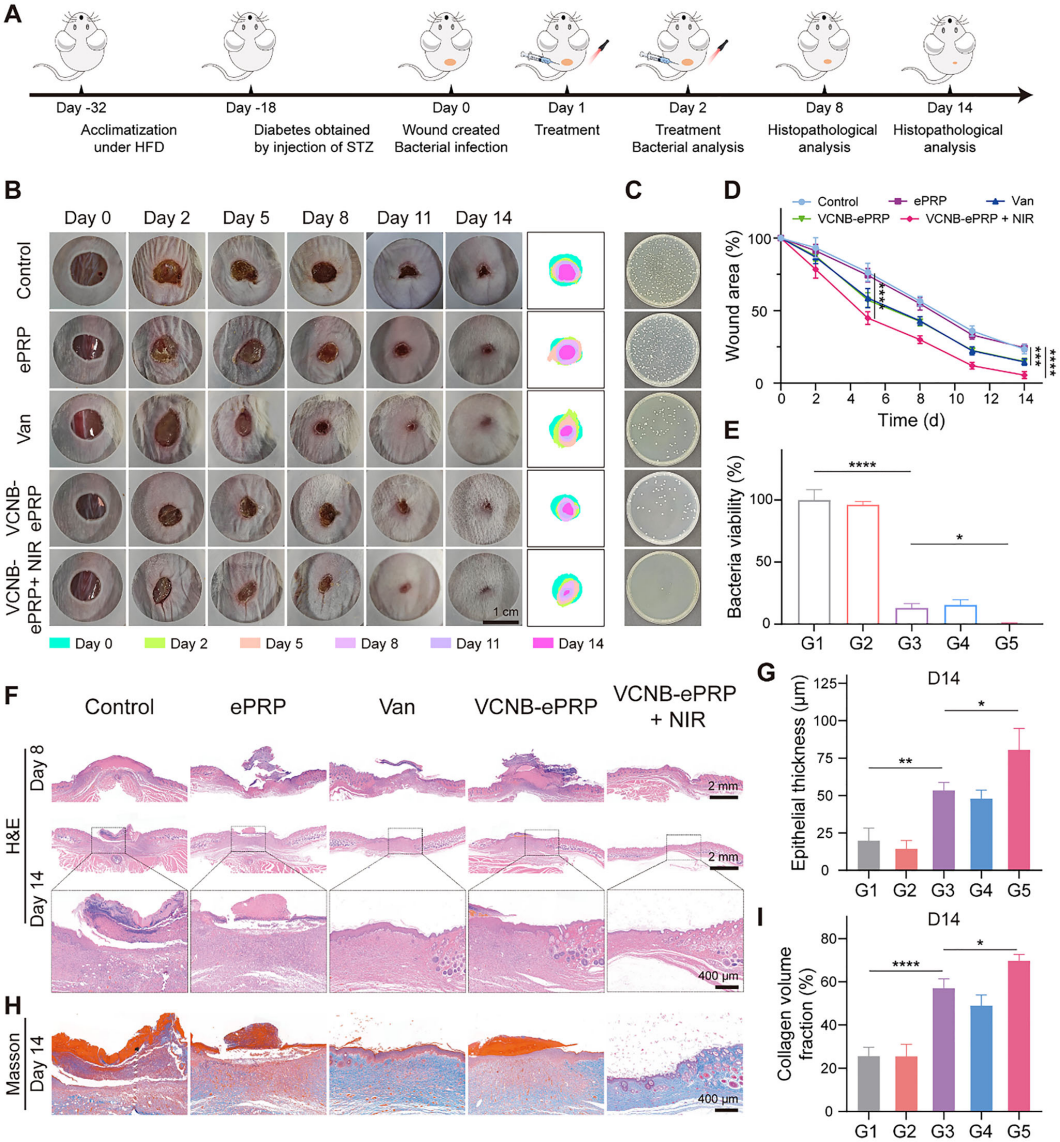
In vivo promotion of MRSA‐infected diabetic wound healing by VCNB‐ePRP. (A) Schematic illustration for the establishment of the MRSA‐infected diabetic wound mouse models and experimental timeline. (B) Representative wound traces and simulations of mice in different groups, and (D) corresponding wound contraction rate (n = 5). (C) Representative photographs of survival bacteria clones on plates in wound tissues on day 2 post‐treatment and (E) corresponding quantitative analysis (n = 3). (F) Representative images of H&E staining of wound tissues on day 8 and day 14 post‐treatment, and (G) epithelial thickness analysis on day 14 (n = 3). (H) Representative of Masson's staining images of wound tissues and (I) quantitative analysis of collagen volume fractions (n = 3). Data are represented as means ± SD. Statistical significance is calculated by one‐way ANOVA with Tukey post hoc test. *p < 0.05, **p < 0.01, ***p < 0.001, ****p < 0.0001, NS means not significant.

The therapeutic approach is depicted in Figure 8A. The successful establishment of diabetic mice was evidenced by the continuous increase in blood glucose value (Figure S19). Following the modeling of MRSA‐infected diabetic wounds with a diameter of 8 mm, mice were randomized into 5 groups (Control, ePRP, Van, VCNB‐ePRP and VCNB‐ePRP + NIR) and received respective treatments on day 1 and day 2. Wound images and quantitative analysis (Figure 8B,D) revealed significantly accelerated healing in the VCNB‐ePRP + NIR group, achieving near‐complete closure (only 5.5% remaining wound area) by day 14, compared to 23.3%, 24.3%, 14.5% and 14.8% in the other groups. Importantly, the VCNB‐ePRP + NIR group demonstrated superior healing efficacy than vancomycin treatment alone. In addition, as revealed in Figure 8C,E, the residual bacterial count in wound tissues of the VCNB‐ePRP + NIR treatment group was significantly lower than all other groups, achieving a 99.4% MRSA clearance rate. These results demonstrated that VCNB‐ePRP could synergistically combine photothermal therapy and Van to enhance bacterial clearance in wounds. Histological evaluation via H&E staining revealed superior tissue regeneration in the VCNB‐ePRP + NIR treatment group, with significantly reduced epidermal gaps and greater epidermal thickness compared to other groups (Figure 8F,G; Figure S20A,B). Furthermore, Masson's trichrome staining on day 14 (Figure 8H,I) showed enhanced collagen deposition in the VCNB‐ePRP + NIR group, with a CVF of 69.6%, markedly higher than in other treatment groups. These results demonstrated that VCNB‐ePRP + NIR accelerated epidermal migration, regeneration and collagen deposition in infected wounds. Collectively, these findings establish that VCNB‐ePRP is able to combine NIR photothermal therapy and Van antibiotics to efficiently eradicate MRSA pathogens in diabetic wound infections, thereby promoting the healing of wounds.

## 3 Conclusions

In summary, we have developed a smart NIR photothermally responsive GFs delivery system based on CNB‐ePRP hydrogel and demonstrated its prominent potential for precisely treating diabetic wounds. Compared with conventional passive‐release hydrogels, the innovative CNB‐ePRP is endowed with in situ activation of PRP and active photothermally‐controlled release of GFs, which significantly improves the bioavailability of GFs in diabetic wounds. The results verify that CNB‐ePRP exhibits multiple potent bioactive properties, including pro‐angiogenic activity, cell proliferation enhancement, ROS‐scavenging capacity, and anti‐inflammatory effects, thereby improving the therapeutic efficacy for diabetic wounds in a mouse model. Moreover, CNB‐ePRP enables loading of vancomycin for multimodal sterilization and efficiently eliminates MRSA, promoting the healing of MRSA‐infected diabetic mouse wounds. Overall, our work presents a promising, versatile PRP‐derived strategy path for diabetic wound treatment and also provides a paradigm for designing other smart bioactive materials.

## 4 Experimental Section

### 4.1 Cell Lines

The human umbilical vein endothelial cells (HUVECs, RRID: CVCL_E5ZU) were purchased from Haixing Biotechnology Co., Ltd (Cat NO: TCH‐C406, Suzhou, China). The mouse mononuclear macrophage cell lines (RAW264.7, RRID: CVCL_0493) were purchased from Procell Life Science & Technology Co., Ltd (Cat NO: CL‐0675, Wuhan, China). The NCTC clone 929 mouse fibroblast cells (L929s, RRID: CVCL_0462) were purchased from CytoNiche Biotechnology Co., Ltd (Cat NO: YC‐C091, Guangzhou, China). All cell lines were routinely tested and confirmed to be contamination‐free by the providers (Haixing Biotechnology Co., Ltd; Procell Life Science & Technology Co., Ltd; CytoNiche Biotechnology Co., Ltd).

### 4.2 Animal Study and Establishment of Animal Models

All animal experimental protocols were approved by the Institutional Animal Care and Use Committee (IACUC) of the Animal Experiment Center of Huazhong Agricultural University (Wuhan, China). Male BALB/c mice (6–8 weeks old) were fed a high‐fat/high‐sugar diet and maintained in a SPF environment. The mice were intraperitoneally injected with 100 mg/kg streptozotocin (STZ, dissolved in 0.1 mM sodium citrate buffer, pH 4.2–4.5) for two consecutive days. Blood glucose levels were monitored daily. After the first injection of STZ, mice demonstrating elevated blood glucose concentrations (≥16.7 mM) for 14 consecutive days were considered diabetic and used for subsequent experiments. Under isoflurane anesthesia conditions, a 10 mm full‐thickness sterile skin wound was created in the midline of the mouse back using a biopsy punch to establish diabetic wound models. Additionally, a skin wound (8 mm diameter) was created and topically inoculated with 20 µL of MRSA suspension (10^8^ CFU/mL), followed by covering with a 3 M dressing. The wound was considered a diabetic infected wound model 24 h post‐inoculation.

### 4.3 Statistical Analysis

Statistical analyses were conducted using GraphPad Prism 8.0.2, and data were presented as mean ± standard deviation (SD). Statistical significance of data was calculated by one‐way ANOVA with Tukey's multiple comparisons test. A significance level of p < 0.05 was considered statistically significant (*p < 0.05, **p < 0.01, ***p < 0.001, ****p < 0.0001, and NS represented no significant difference).

Methods and any associated references are available in the Supporting Information.

## Conflicts of Interest

The authors declare no conflicts of interest.

## Supporting information




**Supporting File**: advs74541‐sup‐0001‐SuppMat.docx.

## Data Availability

The data that support the findings of this study are available from the corresponding author upon reasonable request.
