# Supplementary material for: A Photothermal‐Responsive PRP‐Loaded Hydrogel Engineered With Composite Nanobottle for Controllable Delivery of Growth Factors and Multifunctional Therapy of Diabetic Wounds
Source: Adv Sci (Weinh). 2026 Feb 24;13(25):e22427. doi: 10.1002/advs.202522427 (PMC13137842; doi:10.1002/advs.202522427)
Supplement: Supplementary file 1 — Supporting File: advs74541‐sup‐0001‐SuppMat.docx. [file ADVS-13-e22427-s001.docx]

*Supporting Information*

*of*

**A Photothermal-Responsive PRP-Loaded Hydrogel Engineered with Composite Nanobottle for Controllable Delivery of Growth Factors and Multifunctional Therapy of Diabetic Wounds**

Wen-Qiang Qu^a,†^, Hui-Yun Gu^a,†^, Si-Min Zeng^b^, Chang-Jiang Liu^a^, Xiao Yan^b^, Ting Pan^b^, Chao Jian^a^, Xian-Zheng Zhang^a,b,*^ and Ai-Xi Yu^a,*^

^a^ Dr. W. Q. Qu, Dr. H. Y. Gu, C. J. Liu, Dr. C. Jian, Prof. Dr. X. Z. Zhang, Prof. Dr. A. X. Yu

Department of Orthopedic Trauma and Microsurgery

Zhongnan Hospital of Wuhan University

Wuhan 430071, P. R. China

^b^ Dr. S. M. Zeng, X. Yan, T. Pan, Prof. Dr. X. Z. Zhang

Key Laboratory of Biomedical Polymers of Ministry of Education

& Department of Chemistry

Wuhan University

Wuhan 430072, P. R. China

* Corresponding authors. E-mails: yuaixi@whu.edu.cn (A. X. Yu); xz-zhang@whu.edu.cn (X. Z. Zhang)

†W. Q. Qu and H. Y. Gu contributed equally to this work

**Experimental section**

**Materials.** Polystyrene nanospheres (PS, 2.5%, w/v) with an average diameter of 500 nm and dopamine hydrochloride (98%) were purchased from Macklin Biochemical Technology Co., Ltd (Shanghai, China). Sodium dodecyl sulfate (SDS), toluene (99.5%), tetrahydrofuran (THF, 99.5%) and calcium chloride (CaCl_2_) were purchased from Sinopharm Chemical Reagent Co., Ltd (Shanghai, China). Ethanol (99.8%) was purchased from Chengdu Kelong Chemical Co., Ltd (Chengdu, China). Tris-HCl buffer (10 mM, pH=8.5), gelatin (type B) and thrombin (200 IU mg-1) were purchased from Yuanye Biotechnology Co., Ltd (Shanghai, China). Vancomycin was purchased from Aladdin Biochemical Technology Co., Ltd (Shanghai, China). Calcein AM, PI, CCK-8, LPS、INF-γ, protein quantitation kit (BCA assay), DCFH-DA probe and H_2_O_2_ assay kit were supplied by Beyotime Biotechnology Co., Ltd (Shanghai, China). Mouse PDGF, VEGF, TGF-β, TNF-α, IL-1β, IL-6, IL-10 ELISA kits were supplied by Multisciences Biotechnology Co., Ltd (Hangzhou, China). Anti-CD86-APC and anti-CD206-FITC were purchased from BioLegend (San Diego, USA).

**Synthesis of PDAB nanoparticles.** In brief, 0.5 mL of PS suspension was centrifuged (8000 rpm, 5 min) and washed with ultrapure water for three times. Then, the PS sediment was redispersed in 10 mL of Tris-HCl buffer (10 mM, pH = 8.5) containing dopamine hydrochloride (1 mg/mL) and stirred for 12 h at room temperature. Afterward, the black deposits were collected by centrifugation (6000 rpm, 5 min) and redispersed in 10 mL of Tris-HCl buffer containing dopamine hydrochloride (1 mg/mL) for another round of reaction. After stirring for 12 h, the solid products were centrifuged and washed twice with ultrapure water. Subsequently, the PS@PDA core-shell nanoparticles were obtained and dispersed in 1 mL of ultrapure water. Next, “0.1 mL of toluene” + “9.9 mL of water containing 1% (w/v) sodium dodecyl sulfate” were mixed and sonicated to prepare a 1% (v/v) toluene/water emulsion. Then, 0.5 mL of PS@PDA nanoparticles was dispersed into 10 mL of the toluene/water emulsion and incubated on a shaker (300 rpm, room temperature) for 6 h to swell the PS core. The swelling process was quenched by adding 20 mL of ethanol. After centrifugation (6000 rpm, 5 min) and washing with ethanol, the solid products were incubated with 10 mL of tetrahydrofuran on a shaker (300 rpm, room temperature) for at least 4 h to remove residual PS. Finally, the obtained PDAB nanoparticles were washed twice with tetrahydrofuran and five times with ultrapure water, then dispersed in ultrapure water.

**Fabrication of CNB and Van-CNB nanoparticles.** Gelatin (150 mg) was mixed with 0.5 mL of PBS and incubated at 50 ℃ for 20 minutes to obtain a 30% gelatin solution. Then, 0.5 mL of PBS mixture containing 20 mg of thrombin and 5 mg of PDAB was added, followed by oscillation (300 rpm) at 38 ℃ for 20 min. The precipitate was collected by centrifugation and washed twice with PBS at 38 ℃, and then incubated in an ice bath for 30 minutes to induce the gelation of the solution. Next, the gelation products were re-dispersed by sonication in cold water (0 ℃) for 10 s. Then, the solid products were centrifuged (12000 rpm, 10 min) and washed twice with cold water to obtain thrombin-gelatin-loaded PDAB nanoparticles (donated as composite nanobottle, CNB). The CNB suspension was stored at 4 ℃ for further use. Vancomycin-loaded nanobottles (Van-CNB) were prepared by the same method with an additional 10 mg of vancomycin in the above PBS mixture containing thrombin and PDAB.

**Fabrication of PRP, ePRP, CNB-ePRP and Van-CNB-ePRP hydrogels.** Blood was collected from male SD rats and mixed with ACD-A anticoagulant (1 mL ACD-A: 9 mL blood). The blood was centrifuged at 200 g for 15 min (4 ℃), and the supernatant was collected. After centrifuging at 800 g for 20 min, the upper plasma and the precipitated platelets were collected. The platelets were resuspended in the plasma and concentrated to obtain a PRP solution (the platelet concentration was more than 2×10^9^ mL^-1^). The activator of PRP (donated as activator A) containing 31.9 IU/mL thrombin and 2.5 wt% calcium chloride was prepared by mixing 50 IU of thrombin with 1 mL of calcium chloride. When PRP gel was used alone, the activator A was added (100 µL activator A: 1 mL PRP). Additionally, 1 mL of SA solution (20 mg/mL) was mixed with 1 mL of PRP to obtain the ePRP solution, and then 200 µL of the activator A was added to form the ePRP gel. Then, 100 µL of 2.2 mg/mL CNB (or Van-CNB) was mixed with 100 µL of calcium chloride (5 wt%) at 4 ℃ to form the activator B. The activator B was mixed with ePRP solution (200 µL activator B: 2 mL ePRP) and mixed well to obtain CNB-ePRP gel and Van-CNB-ePRP gel.

**Characterization of nanoparticles and hydrogels.** The size and zeta potential of nanoparticles were measured using a Nano-ZS ZEN3600 device (Malvern Instruments). The scanning electron microscope (SEM) images of nanoparticles were obtained using a scanning electron microscope (Zeiss Sigma, UK). The transmission electron microscope (TEM) images of nanoparticles were obtained using a JEM-2100Plus spectroscopy (JEOL, Japan). The presence of thrombin in CNB nanoparticles was detected by SDS-PAGE, and the drug loading efficiency of thrombin in CNB was determined using a BCA assay kit. The drug loading capacity of Van-CNB was analyzed using a UV-Vis spectrophotometer (Lambda Bio40). The SEM images of hydrogels were obtained by a Tescan MIRA microscope (TESCAN, Czech Republic) and the corresponding energy dispersive spectroscopy (EDS) elements were analyzed using an Ultim Extreme device (UK). The rheological mechanical properties of hydrogels were assessed by a TA DHR-2 rheometer (TA Instruments, USA). The photothermal performance of nanoparticles and hydrogels was conducted using an 808 nm laser (LOS-BLD-0808-005W, China). Temperature changes were recorded by a JIR-A384 thermal camera. The cell viability assay was measured using a SpectraMax 190 microplate reader (USA). Inverted fluorescence imaging was conducted with an OLYMPUS U-HGLGPS IX73PRF microscope (Japan). A BD flow cytometer (USA) was used for flow cytometry analysis. Routine blood tests were measured with an MC-6200VET analyzer (China) and blood biochemistry tests were assessed using an automatic blood analyzer (MNCHIP, China).

**The photothermal effect of PDAB and CNB nanoparticles.** Firstly, 1 mL of PDAB solution and CNB solution were irradiated with an 808 nm laser (1.0 W cm^-2^, 10 min) at different concentrations (0, 50, 100, 200 μg mL^-1^) and different laser irradiation powers (0.5, 0.75, 1.0, 1.5 W cm^-2^). The temperature changes were recorded using a JIR-A384 thermal camera. Furthermore, the photothermal conversion stabilities of PDAB and CNB were evaluated for 3 heating/cooling cycles.

**NIR-triggered release of thrombin from CNB nanoparticles.** First, 1 mL of CNB solution (100 μg/mL) was exposed to three different conditions (20 ℃, 37 ℃ and 808 nm irradiation with 1.0 W cm^-2^ power) for 10 min. Then, the supernatant was collected and the concentration of the released thrombin was detected with BCA assays.

**Extracellular antioxidant activity evaluation of CNB nanoparticles.** The ROS scavenging capacity of CNB was evaluated using H_2_O_2_ scavenging essay, 2,2'-azinobis-3-ethylbenzthiazoline-6-sulphonate (ABTS) essay, 1,1-diphenyl-2-picrylhydrazyl (DPPH) essay and tetramethylbenzidine (TMB) essay. The H_2_O_2_ scavenging capacity was determined by mixing 2 mL of H_2_O_2_ (1 mM) with the CNB. After 15 min, 50 μL of the supernatant was obtained and measured using the H_2_O_2_ assay kit. In the ABTS essay, 3 mg of ABTS was incubated in 0.5 mL of ultrapure water containing potassium persulfate (1 mg) for 12 h to generate radicals. Subsequently, 5 µL of the radical ABTS solution, CNB nanoparticles (40 µL, 5 mg/mL), and 955 µL of ultrapure water were mixed and incubated in the dark for 15 min. In the DPPH essay, CNB nanoparticles (40 µL, 5 mg/mL) were incubated with DPPH free radical (0.1 mM, 1 mL) in the dark for 30 min. In the TMB essay, CNB nanoparticles (40 µL, 5 mg/mL) were incubated with TMB (0.1 mM), ferrocene-carboxylic acid (0.1 mM), and H₂O₂ (1 mM) in the dark for 15 min. Finally, the absorbances of the above samples were tested by a UV-Vis spectrophotometer.

**In vitro degradation evaluation of hydrogels**

Briefly, 1 mL of different hydrogels (PRP and CNB-ePRP ) was prepared and added to centrifuge tubes containing 10 mL of serum-free DMEM medium (pH 7.4), followed by keeping in a shaker (100 rpm, 37 ℃). At specific time intervals, the remaining hydrogel in the tubes was removed and the remaining hydrogel masses were measured.

**NIR-triggered total protein, growth factor and vancomycin release from hydrogels.** First, 500 µL of PRP, 1000 µL of CNB-ePRP and 1000 µL of CNB-ePRP were respectively placed in centrifuge tubes containing 5 mL of PBS for vibration (150 rpm, 37 ℃). Then, 500 µL of the supernatant was collected and replaced with an equal volume of fresh PBS at specific interval times (0, 1, 3, 6, 9, 12, 24, 48, 72 h). The CNB-ePRP group was irradiated with or without 808 nm near-infrared (NIR) laser (1.0 W/cm²) for 5 min at 0, 1, 3, 6, 9, 12 and 24 h. The concentrations of the released total protein and growth factors such as PDGF, VEGF, TGF-β and EGF from PRP or CNB-ePRP were evaluated using a BCA kit and ELISA kits. Similarly, the assay of the NIR-responsive release of vancomycin from Van-CNB-ePRP was conducted and analyzed using a UV-Vis spectrophotometer.

**Cell lines and cell culture.** The human umbilical vein endothelial cells (HUVECs, RRID: CVCL_E5ZU) were purchased from Haixing Biotechnology Co., Ltd (Cat NO: TCH-C406, Suzhou, China). The mouse mononuclear macrophage cell lines (RAW264.7, RRID: CVCL_0493) were purchased from Procell Life Science & Technology Co., Ltd (Cat NO: CL-0675, Wuhan, China). The NCTC clone 929 mouse fibroblast cells (L929s, RRID: CVCL_0462) were purchased from CytoNiche Biotechnology Co., Ltd (Cat NO: YC-C091, Guangzhou, China). All cell lines were routinely tested and confirmed to be contamination-free by the providers (Haixing Biotechnology Co., Ltd; Procell Life Science & Technology Co., Ltd; CytoNiche Biotechnology Co., Ltd). HUVECs cells were cultured in Dulbecco’s modified Eagle’s medium (DMEM) medium supplemented with 10% fetal bovine serum (FBS) and 1% penicillin/streptomycin (100 U/mL). RAW264.7 cells were cultured in DMEM medium supplemented with 10% heat-inactivated FBS and 1% penicillin/streptomycin. L929 cells were cultured in RPMI-1640 medium. All cells were incubated at 37 ^o^C with a 5% CO_2_ atmosphere.

**Bacteria culture.** Methicillin-resistant Staphylococcus aureus (MRSA, ATCC 43300) was used for antimicrobial studies. MRSA on solid Luria-Bertani (LB) agar plates were separated and added into fresh LB broth. Then, MRSA suspension was incubated in a shaking incubator (200 rpm) at 37 ℃. The bacterial solution was used for subsequent antibacterial experiments when the OD at 600 nm reached 0.1.

**Cell viability and cell proliferation assays.** First, HUVECs cells were used for the cytotoxicity of CNB nanoparticles. HUVECs cells (1 × 10^4^ per well) were plated into 96-well plates and incubated for 24 h. Then, the cells were treated with CNB nanoparticles at different concentrations (0, 10, 20, 50, 100, 200 μg/mL). After incubation for 24 h, the CCK-8 assay was applied to evaluate the viability of HUVECs. Next, HUVECs or L929 cells (1 × 10^5^ cells per well) were placed in the lower chamber of a 6-well transwell (8 μm pore size), while five different groups of materials (Control, SA, ePRP, CNB-ePRP, CNB-ePRP + NIR) were placed in the upper chamber. In the CNB-ePRP + NIR laser group, the hydrogel was regularly irradiated with an 808 nm laser for 5 min at 24 h. To avoid the direct heat effect on cells, a portion of the culture medium in the lower chamber was separated from the cells during irradiation. After cooling for 10 min, the co-cultivation is resumed. After incubation for 24, 48, 72, and 96 h, the cell proliferation was assessed using the CCK-8 assay. In addition, the proliferation of HUVECs was further investigated through live/dead cell staining assays.

**Cell scratch assay.** Scratch assay was performed to detect the HUVECs cells migration after CNB-ePRP treatment. 3 × 10^5^ HUVECs cells were plated into 6-well plates and incubated until almost overgrown. Then, a pipette tip (200 μL) was used to draw several uniformly wide lines in each well to create scratches. The wells were washed carefully using PBS. HUVECs cells were treated with DMEM medium (1% FBS) and five different groups of materials (Control, SA, ePRP, CNB-ePRP, CNB-ePRP + NIR). Afterwards, cell migration was recorded using an inverted microscope at 24 and 48 h.

**Tube formation assay.** Briefly, 250 μL of thawed Matrigel was added to each lower chamber of a 24-well plate, incubated at 37 °C for 1 h. Then, 5 × 10^4^ HUVECs were seeded into the Matrigel-coated lower chamber and treated with various materials. In the NIR laser group, the hydrogel was irradiated with an 808 nm laser for 5 min. HUVECs tube images were recorded by an inverted microscope and analyzed by ImageJ software.

**Intracellular antioxidant activity evaluation.** Briefly, HUVECs (5 ×10^4^ cells per well) were seeded into 24-well plates and incubated for 12 h. Then, the cells were treated with 100 µM H_2_O_2_ plus different materials (PBS, SA, ePRP, CNB-ePRP) for 12 h. Subsequently, the cells were stained with FBS-free DMEM containing fluorescent probe DCFH-DA (10 µM) and Hoechst 33342 (5 μg/mL) for 20 min. After washing three times with FBS-free medium, the intracellular ROS levels of HUVECs were assessed by an inverted fluorescence microscope. Additionally, the proliferative capacity of HUVECs treated with CNB-ePRP hydrogel in an oxidative environment was evaluated using the CCK-8 assay.

**Macrophage-related anti-inflammatory capacity evaluation.** RAW264.7 cells (1 × 10^6^ cells/well) were seeded in 6-well plates and incubated with LPS (100 ng mL^-1^) and IFN-γ (10 ng mL^-1^) for 24 h to induce M1 phenotype polarization. Subsequently, the supernatants were removed and washed three times. Then, the cells were incubated with PBS, SA, ePRP and CNB-ePRP for 24 h. After treatment, the cells were collected and labeled with anti-CD86 and anti-CD206 antibodies for 30 minutes at 4 ℃ in the dark. After centrifugation and washing, the cells were resuspended in PBS and analyzed by flow cytometry. Next, iNOS and CD206 immunofluorescent stainings and RT-qPCR were performed by Servicebio Biotech Co., Ltd., (Wuhan, China). In addition, the levels of TNF-α, IL-6, IL-4, IL-10 and IL-1β in the supernatant were quantified using ELISA kits.

**Antibacterial activity of hydrogels.** First, 1 mL of MRSA suspension (10⁸ CFU/mL) was inoculated in 48-well plates, and was added with different sterile materials (PBS, Van, ePRP, VCNB-ePRP, VCNB-ePRP + NIR). For the Van-CNB-ePRP + NIR group, the samples were irradiated with a near-infrared laser (1.0 W/cm², 5 min) for twice (10 min interval), and all samples were incubated for 6 h. Subsequently, the bacterial suspensions were collected, and the antibacterial efficacy of the VCNB-ePRP hydrogel was evaluated using the dilution plating method, bacterial live/dead staining, and scanning electron microscopy (SEM).

**Animal study and establishment of animal models.** All animal experimental protocols were approved by the Institutional Animal Care and Use Committee (IACUC) of the Animal Experiment Center of Huazhong Agricultural University (Wuhan, China). Male BALB/c mice (6–8 weeks old) were fed a high-fat/high-sugar diet and maintained in a SPF environment. The mice were intraperitoneally injected with 100 mg/kg streptozotocin (STZ, dissolved in 0.1 mM sodium citrate buffer, pH 4.2-4.5) for two consecutive days. Blood glucose levels were monitored daily. After the first injection of STZ, mice demonstrating elevated blood glucose concentrations (≥16.7 mM) for 14 consecutive days were considered diabetic and used for subsequent experiments. Under isoflurane anesthesia conditions, a 10 mm full-thickness sterile skin wound was created in the midline of the mouse back using a biopsy punch to establish diabetic wound models. Additionally, a skin wound (8 mm diameter) was created and topically inoculated with 20 µL of MRSA suspension (10⁸ CFU/mL), followed by covering with a 3M dressing. The wound was considered a diabetic infected wound model 24 h post-inoculation.

**The photothermal effect evaluation of hydrogels at mice wound sites.** After the mouse wound models were established, 50 µL of CNB-ePRP was applied locally at the wound sites. Then, the wounds were irradiated with a NIR laser (1.0 W/cm²) for 5 min, and the temperature changes of the wounds were monitored using a thermal camera.

**Evaluation of diabetic wound healing and histological analysis.** After the diabetic wound model was established, 50 μL of different groups of materials (blank, SA, ePRP, CNB-ePRP, CNB-ePRP + NIR) was applied locally at the mouse wound. The wounds in the group of CNB-ePRP + NIR were irradiated with an 808 laser (1 W/cm^2^, 5 min) on day 0 and day 2. After treatment, wounds were dressed with a 3M dressing and the dressings were changed every two days. The photos of wound healing areas were recorded using a camera on days 0, 2, 5, 8, 11, 14 after treatment. In addition, on days 8 and 14 post-treatment, the wound tissues of all groups were harvested for H&E staining, Masson staining, Ki67 immunohistochemical (IHC) staining, and CD31 immunofluorescence (IF) staining. In addition, on days 8 and 14, these tissue samples were also subjected to IHC stainings (TNF-α and IL-10) and IF stainings (CD86 and CD206) to investigate the immunomodulatory effects of CNB-ePRP gel on diabetic wounds.

**Biocompatibility evaluation *in vivo.*** The blood biochemical tests of the mice were regularly monitored after treatment. At the end of the experiment, the major organs of the mice, such as the heart, liver, spleen, lungs, and kidneys, were harvested for hematoxylin and eosin (H&E) staining.

**Evaluation of infected diabetic wound healing in mice.** After the diabetic infected wound models were established, 50 µL of different groups of materials (blank, ePRP, Van, VCNB-ePRP, VCNB-ePRP + NIR) were applied locally to the wounds. The wounds in the group of VCNB-ePRP + NIR were exposed to an 808 laser (1 W/cm^2^, 5 min) for twice per day (10 min interval) on days 1 and 2. After treatment, the wounds were dressed with 3M dressings, which were changed every two days. The images of wound healing areas were recorded on days 0, 2, 5, 8, 11, 14 post-treatment. On day 2, wound tissues were harvested and analyzed for residual bacterial counts using the dilution plating method. In addition, local wound tissues were subjected to H&E staining and Masson's staining.

**Statistical analysis.** Statistical analyses were conducted using GraphPad Prism 8.0.2, and data were presented as mean ± standard deviation (SD). Statistical significance of data was calculated by one-way ANOVA with Tukey’s multiple comparisons test. A significance level of p < 0.05 was considered statistically significant (*p < 0.05, **p < 0.01, ***p < 0.001, ****p < 0.0001, and NS represented no significant difference).


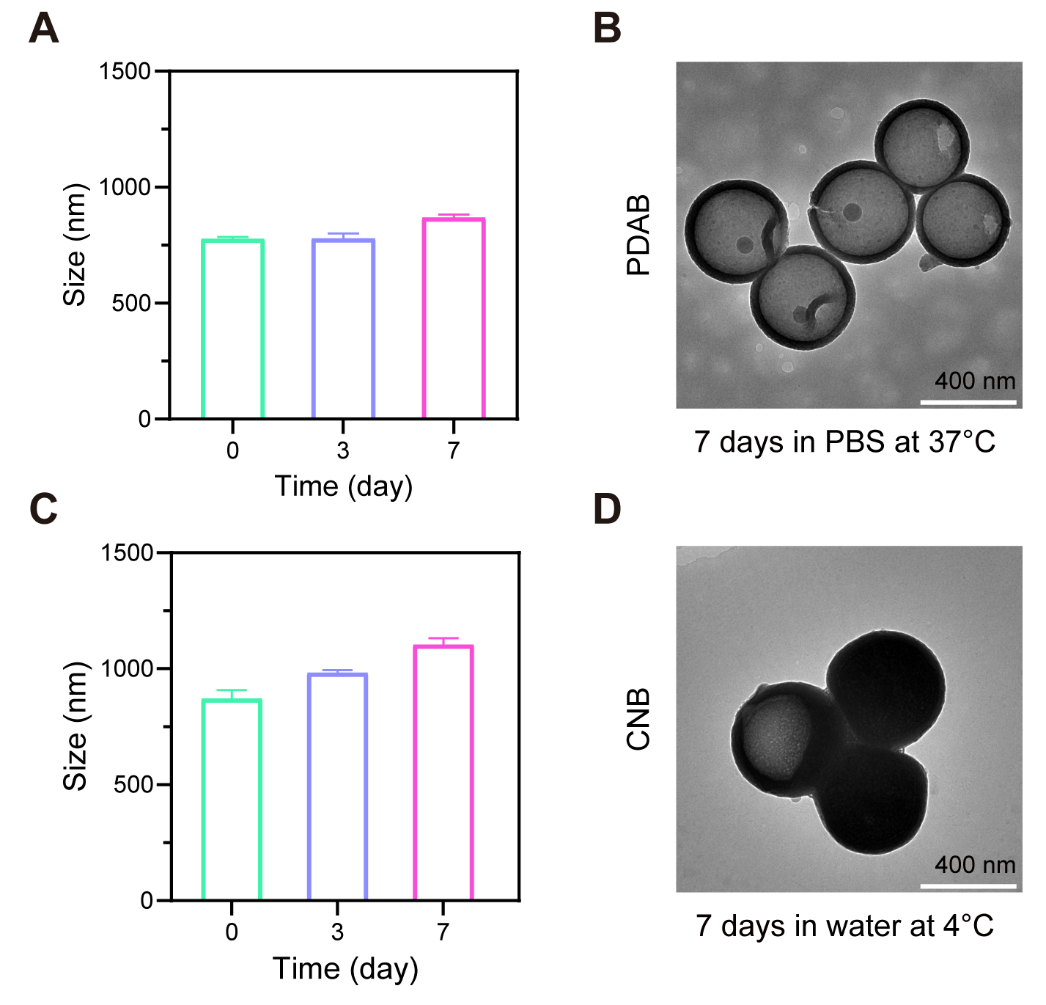


**Figure S1.** Stability characterization of PDAB and CNB nanoparticles. A) Hydrated particle size of PDAB nanoparticles ((n = 3) and B) TEM images after incubation with PBS for 7 days. C) Hydrated particle size of CNB nanoparticles (n = 3) and D) TEM images after incubation with water for 7 days.


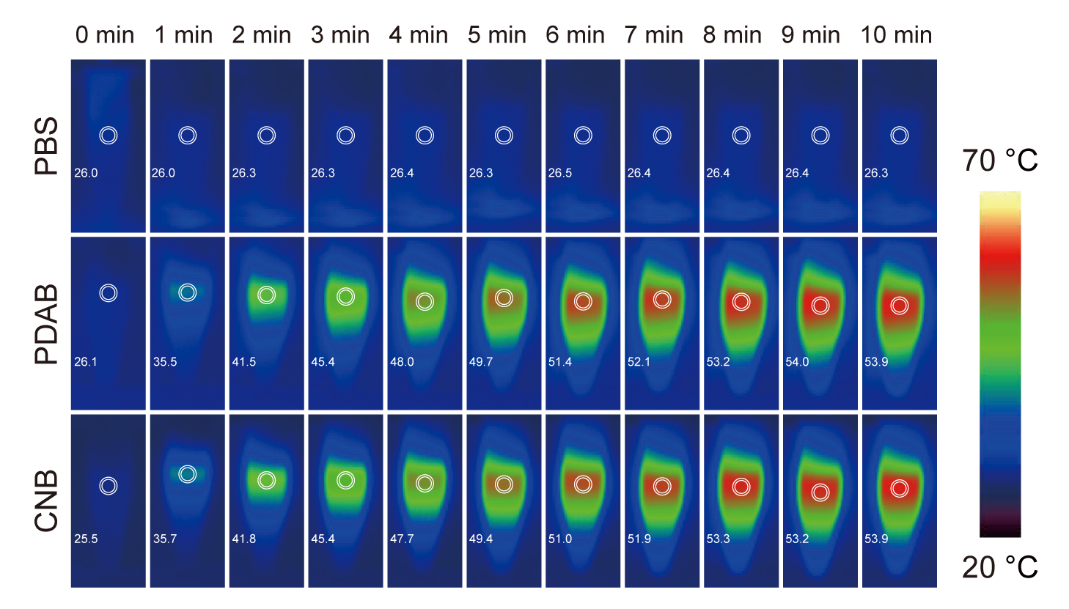


**Figure S2.** Representative photothermal temperature images of PDAB and CNB with diﬀerent concentrations (100 μg mL^-1^) under 808 nm laser irradiation (1.0 W cm^-2^) for 10 min.


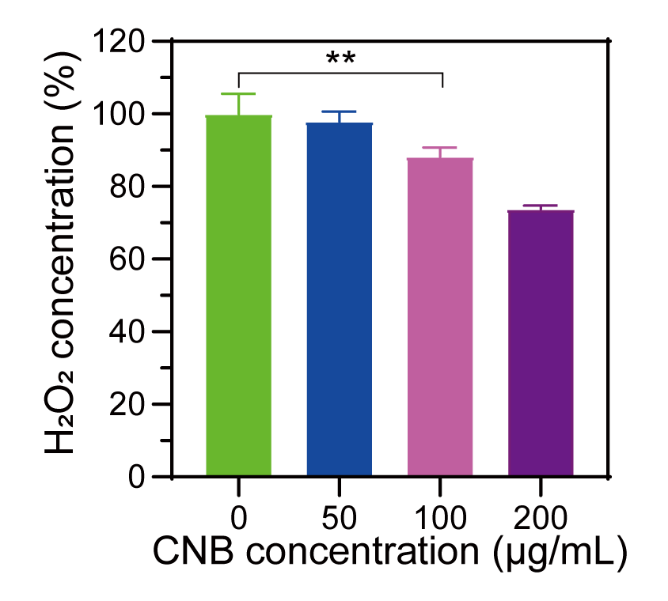


**Figure S3.** Analysis results of the clearance efficiency of H_2_O_2_ for CNB nanoparticles at different concentrations (n = 3).


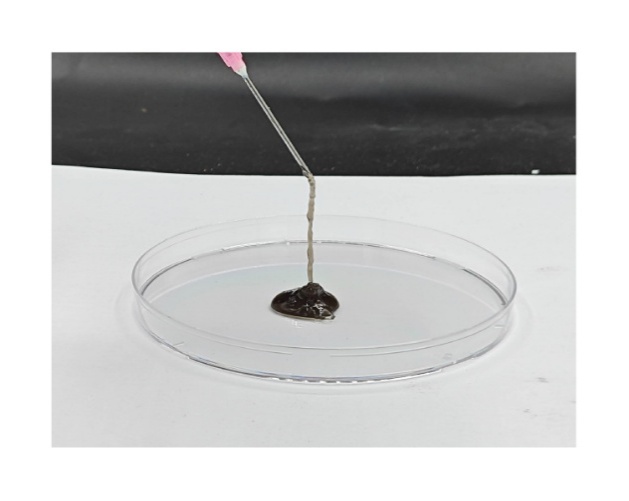


**Figure S4.** Injection image of the CNB-ePRP gel.


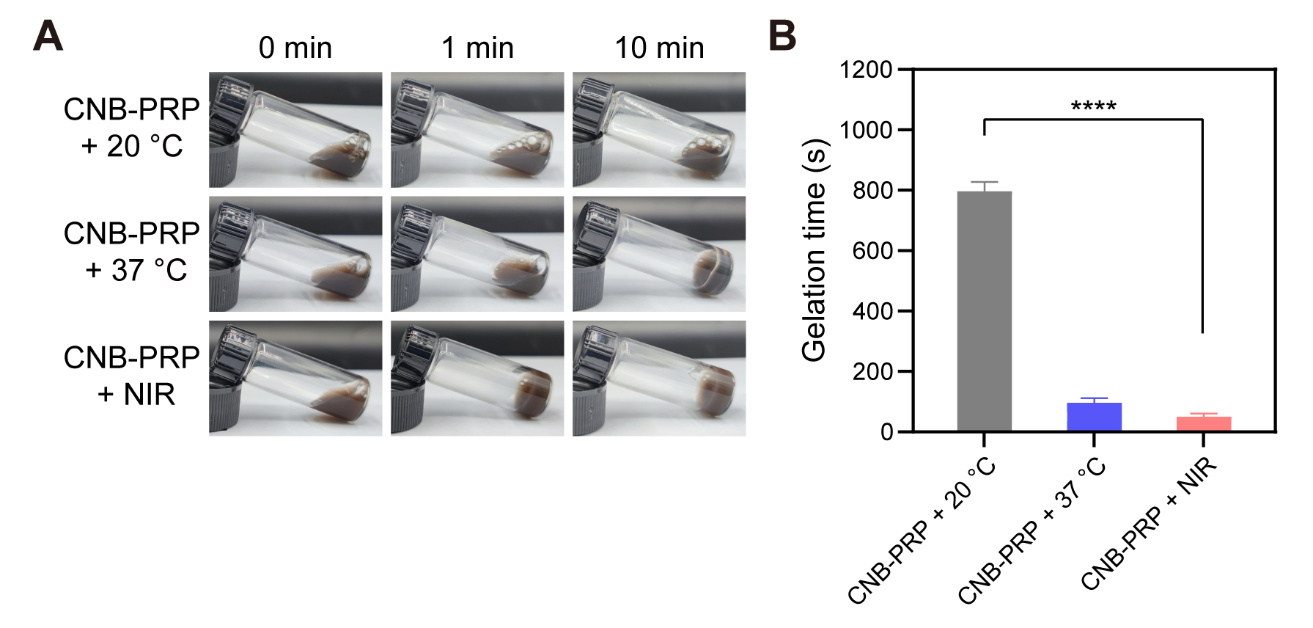


**Figure S5.** A) Representative images of gelation for CNB-PRP controlled by NIR irradiation and B) quantification analysis of gelation times (n = 3). Data are represented as means ± SD. Statistical significance is calculated using Student's t-test analysis. *p < 0.05, **p < 0.01, ***p < 0.001, ****p < 0.0001.


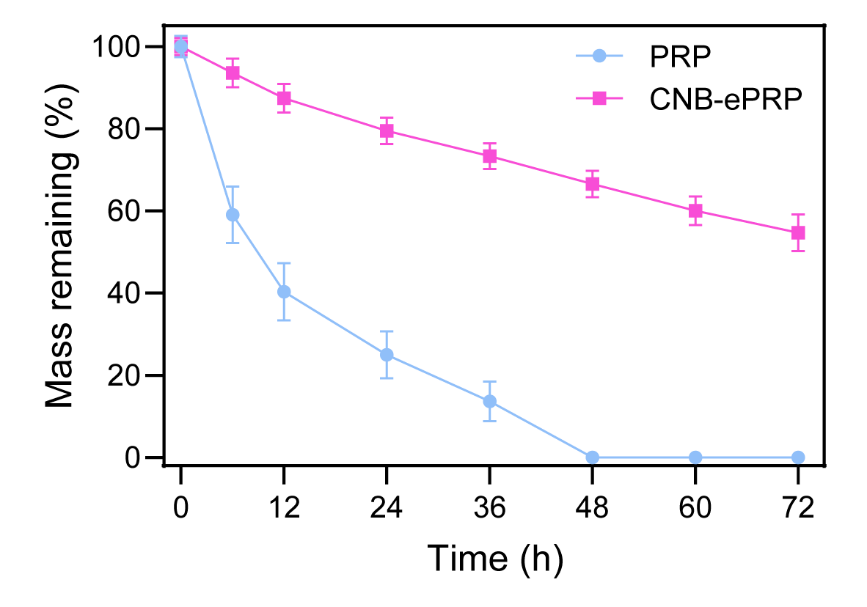


**Figure S6.** Degradation test results of PRP and CNB-ePRP gels (n = 3).


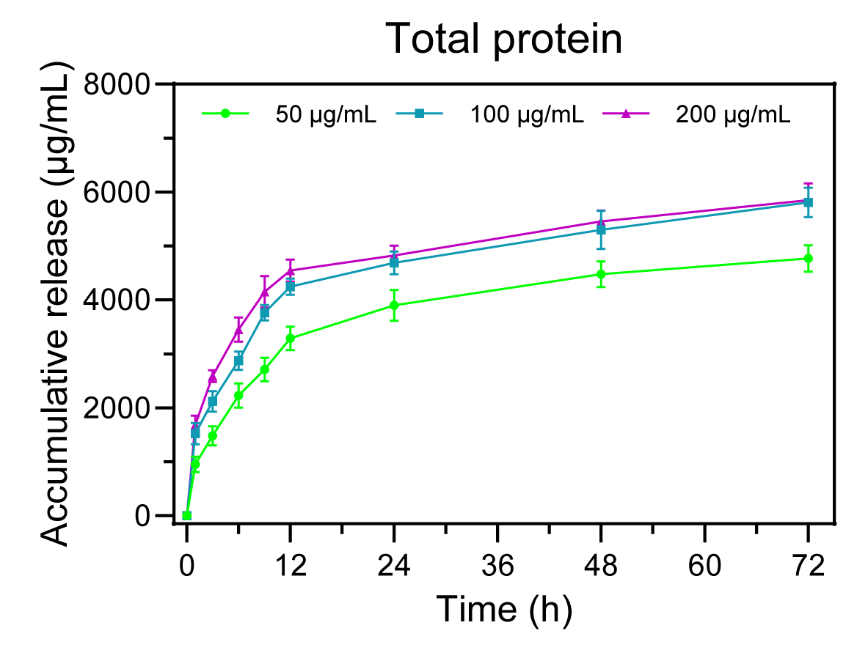


**Figure S7.** Total protein release profiles of CNB-ePRP gel with different concentrations of CNB nanoparticles (n = 4).


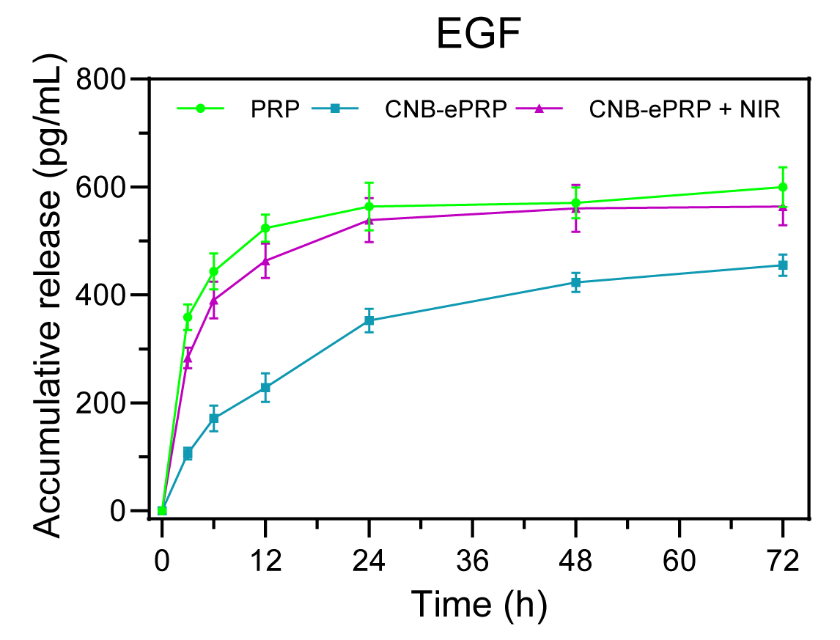


**Figure S8.** EGF release profiles of CNB-ePRP gel with or without NIR irradiation (1.0 W cm^-2^, n = 4).


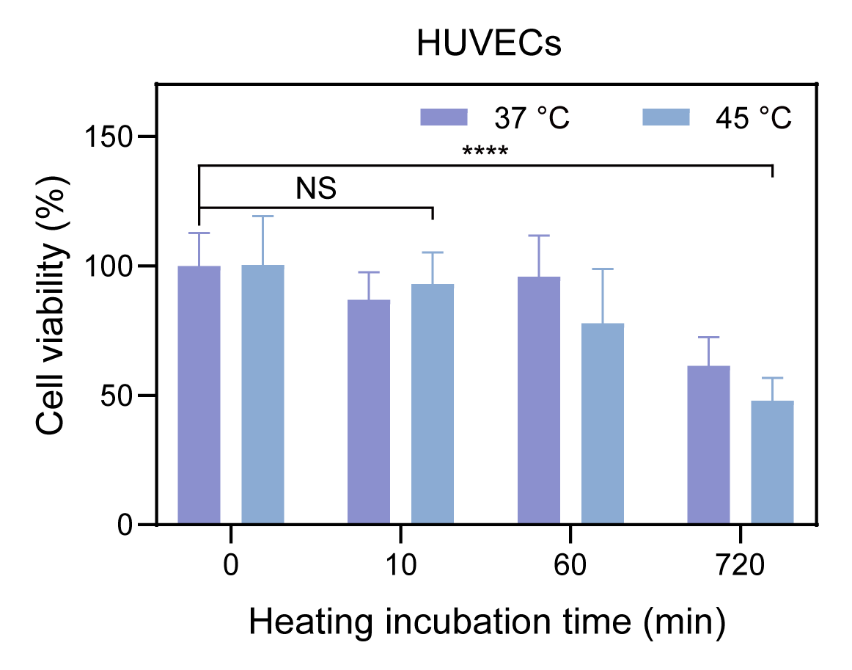


**Figure S9.** Effect of thermal incubation on VEGF-induced proliferative activity of HUVECs cells (CCK-8 assay, n = 6).


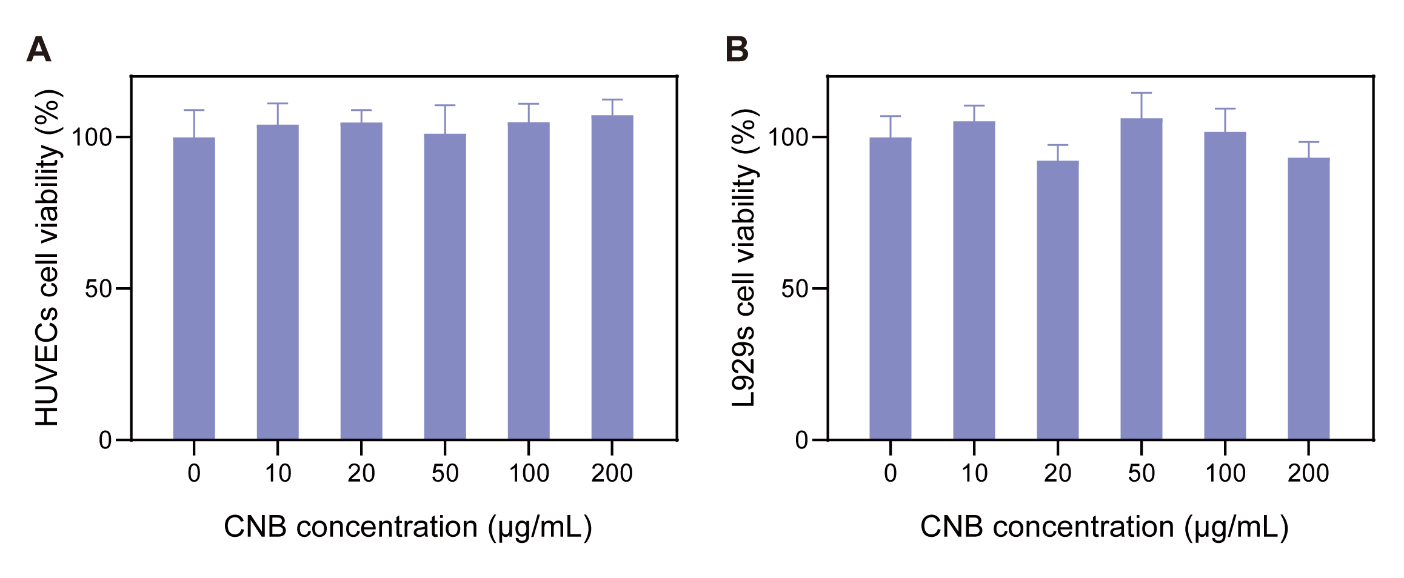


**Figure S10.** A, B) Cell viability analysis of CNB nanoparticles in CNB-ePRP gel on HUVECs and L929s cells using CCK-8 assays (n = 4).


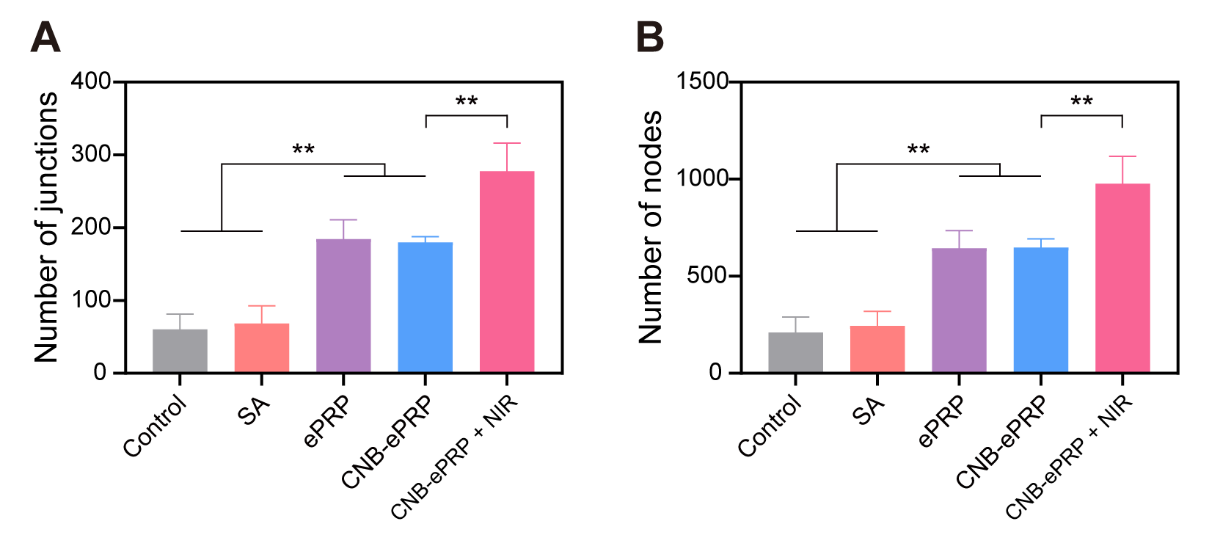


**Figure S11.** Supplementary results of HUVECs tube formation assay after treatment with PBS, SA, ePRP, CNB-ePRP, CNB-ePRP + NIR. A) Number of junctions, and B) number of nodes (n = 3).


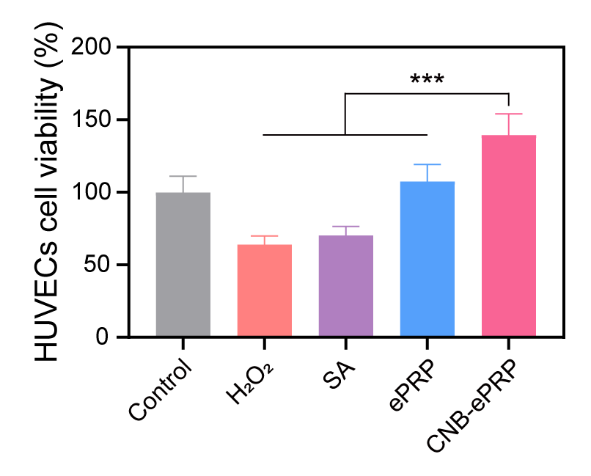


**Figure S12.** CCK-8 analysis for HUVECs after culturing in an oxidative stress environment (100 μM H_2_O_2_; n = 5).


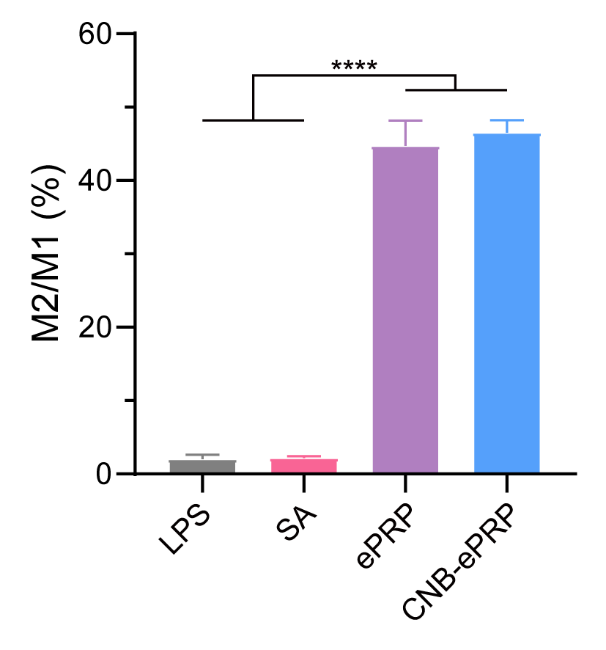


**Figure S13.** Quantitative analysis of M1/M2 macrophages for flow cytometry (n = 4). Data are represented as means ± SD. Statistical significance is calculated by one-way ANOVA with Tukey post hoc test. *p < 0.05, **p < 0.01, ***p < 0.001, ****p < 0.0001, NS means not significant.


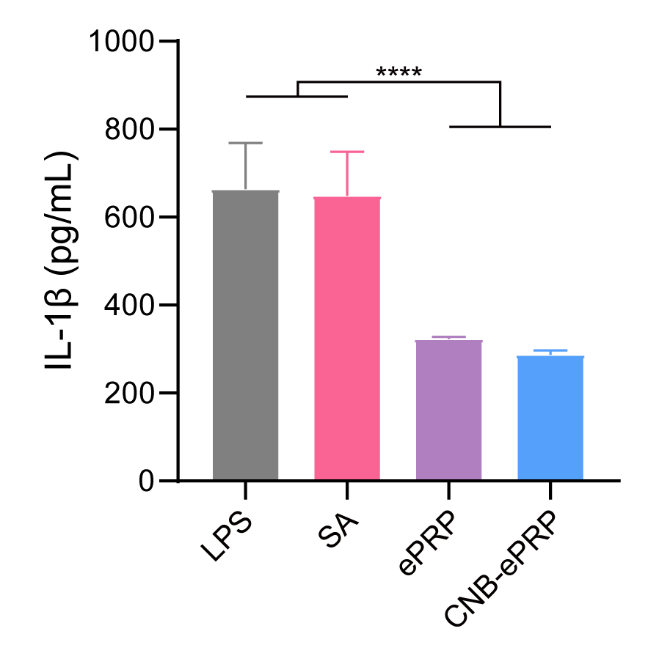


**Figure S14.** IL-1β analysis for RAW264.7 cells after treatment, measured by ELISA (n = 4).


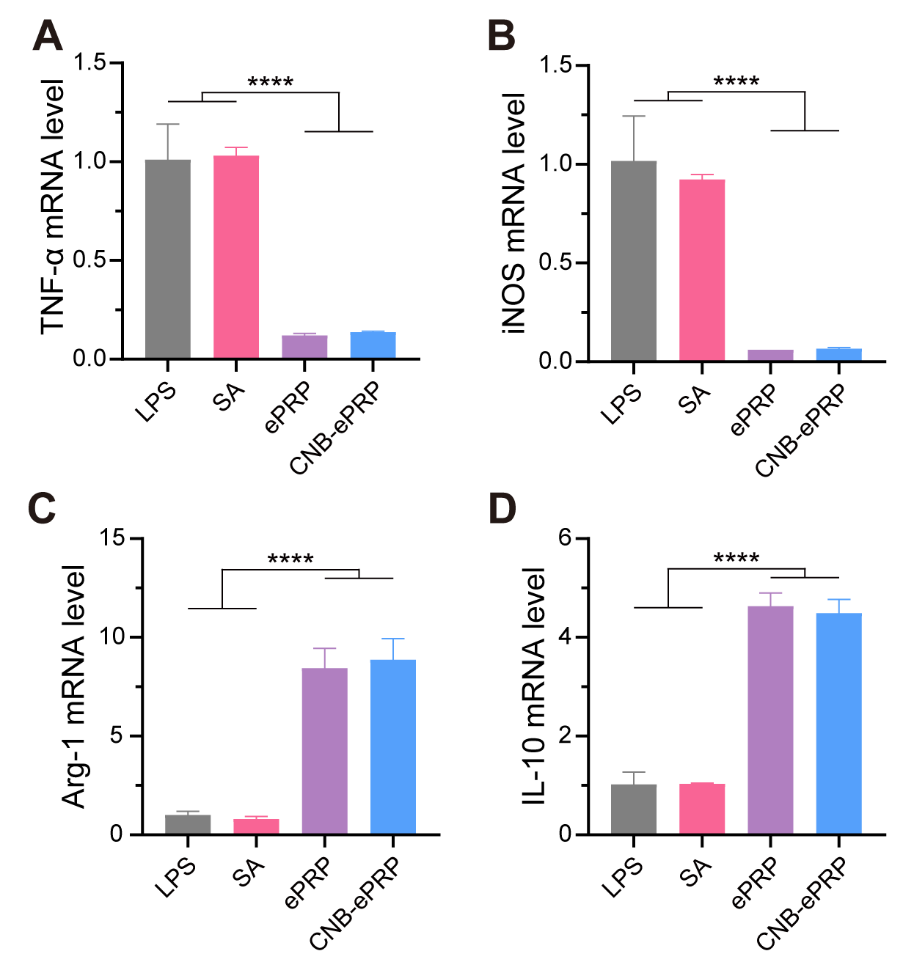


**Figure S15.** The relative mRNA levels of A) TNF-α, B) iNOS, C) Arg-1 and D) IL-10 of macrophages in all groups (n = 3).


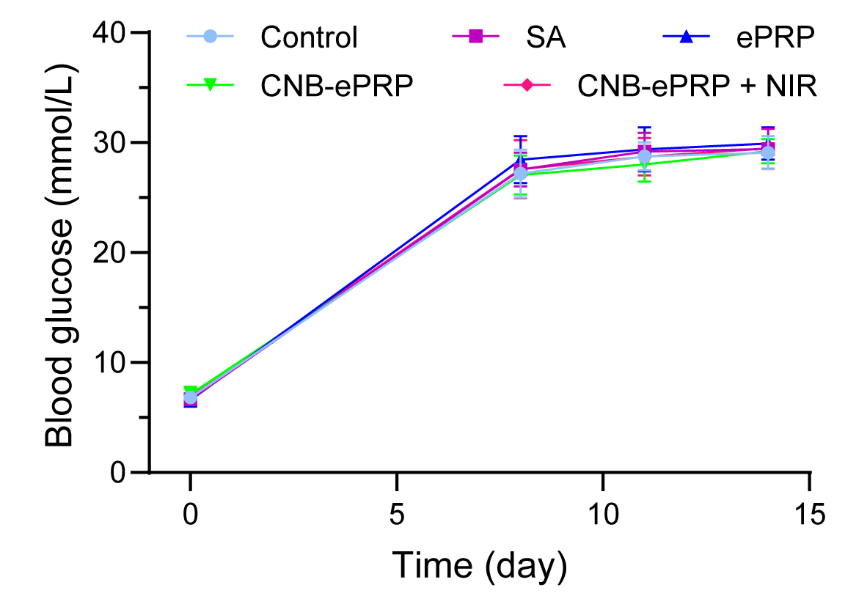


**Figure S16.** Results of blood glucose monitoring in mice (establishment of diabetic wound model), while day 0 represents the day of STZ injection (n = 8).


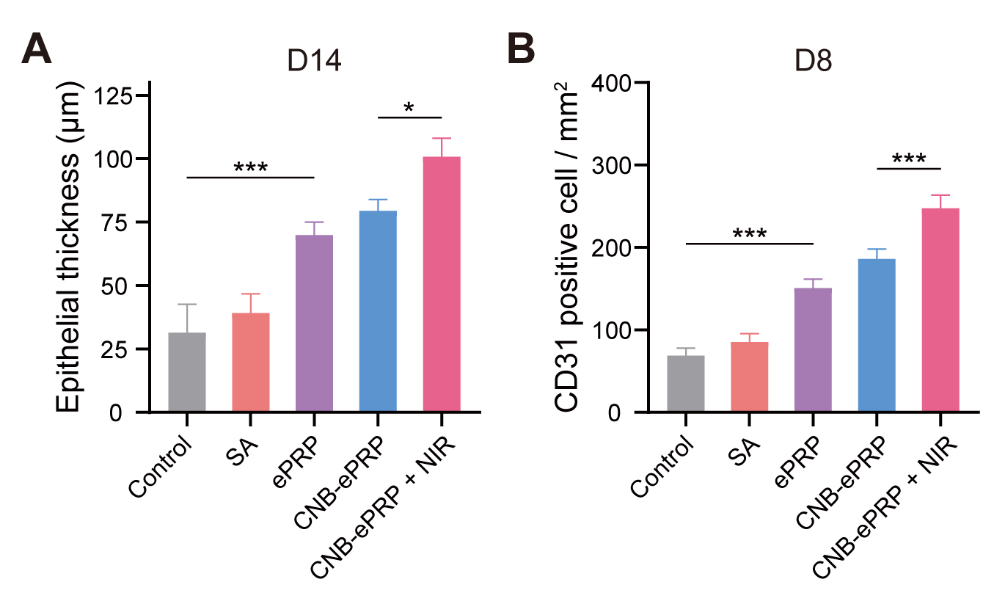


**Figure S17.** A) Quantitative analysis of epithelial thickness for wounds on day 14 and B) IF quantitation analysis of CD31-labeled cells on day 8 (n = 3).


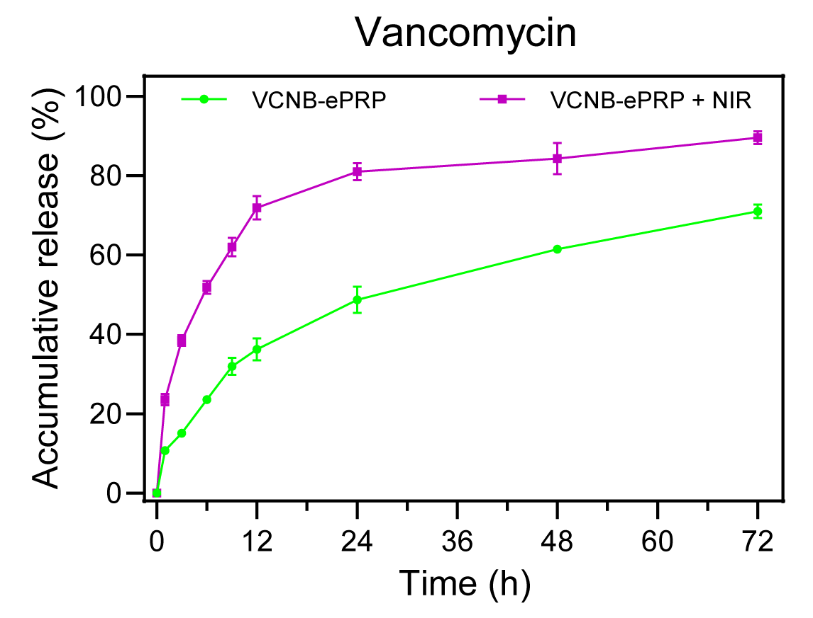


**Figure S18.** Vancomycin release profiles of VCNB-ePRP gel with or without NIR irradiation (1.0 W cm^-2^, n = 3).


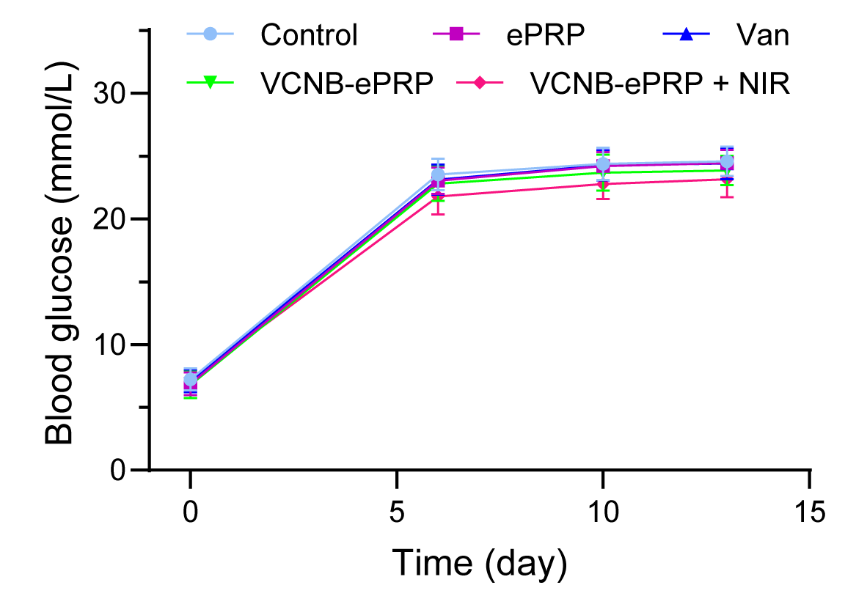


**Figure S19.** Results of blood glucose monitoring in mice (establishment of MRSA-infected diabetic wound model), while day 0 represents the day of STZ injection (n = 8).


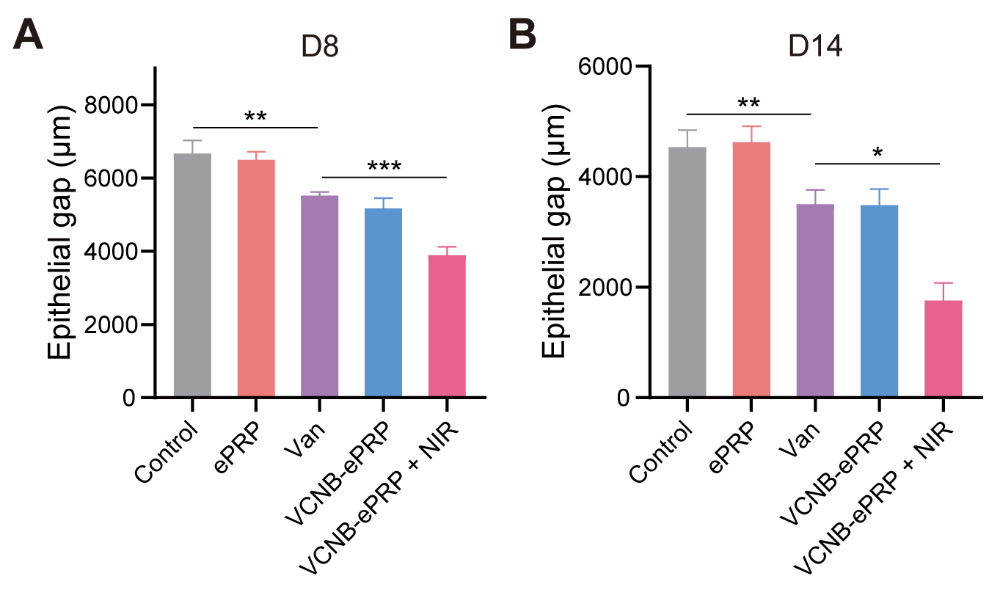


**Figure S20.** A, B) Epithelial gap analysis of wounds on day 8 and day 14 post-treatment, respectively (n = 3).

**Table S1.** The corresponding element atomic analysis of the SEM mapping of CNB-ePRP hydrogel

| Element | Element Atomic (%) |
| --- | --- |
| C | 64.68 |
| O | 34.24 |
| S | 0.28 |
| Ca | 0.80 |
